# Spatial behavior in rehabilitated orangutans in Sumatra: Where do they go?

**DOI:** 10.1371/journal.pone.0215284

**Published:** 2019-05-01

**Authors:** Dominik Fechter, Simone Ciuti, Doris Kelle, Peter Pratje, Carsten F. Dormann, Ilse Storch

**Affiliations:** 1 Freiburg University, Wildlife Ecology and Management, Freiburg, Germany; 2 Laboratory of Wildlife Ecology and Behaviour, School of Biology and Environmental Science, University College Dublin, Science West, Belfield, Dublin, Ireland; 3 Frankfurt Zoological Society, Pematang Sulur, Telanaipura, Jambi/Indonesia; 4 Freiburg University, Biometry and Environmental System Analysis, Freiburg, Germany; University of Sydney, AUSTRALIA

## Abstract

Wildlife restoration is one of the key components of conservation strategies, and this includes the rehabilitation and release of animals confiscated from wildlife traffickers. When primates are re-introduced, most individuals need a pre-release training to acquire the skills needed to survive in the wild. Pre-release training may either negatively or positively affect primate post-release behavior and survival. Post-release behavior, however, has rarely been monitored even though it is the only means to assess the survival of released individuals. Here, we present a thorough analysis of data from a 3-year radio tracking study on 32 orangutans (*Pongo abelii*) released in Sumatra after their rehabilitation. We investigated whether and how the age at release, the duration of the pre-release rehabilitation and training, and the release location affected the post-release individual spatial behavior. Orangutans released at older age exhibited post-release habitat selection patterns that were more comparable to that shown by wild conspecifics, i.e., they chose areas closer to rivers and at lower elevations (150–250 meters a.s.l.) where previous research had documented greater food availability. In contrast, individuals released at younger age showed a stronger spatial dependency on the rehabilitation station and exhibited disrupted habitat selection patterns; although after several months after the release all individuals tended to decrease their spatial reliance on the rehabilitation facility. This study indicates that the rehabilitation of individuals for a longer period and their release further from the rehabilitation station have facilitated the subsequent development of more natural spatial behavior, i.e. driven by food availability rather than by the dependence on care-giving human facility. Our study provides indications on how to improve the rehabilitation and release of confiscated orangutans, highlighting the importance of the age at release, the length of the rehabilitation program, and the location of the release site.

## Introduction

Population restorations are an important tool used to foster viable populations of threatened species worldwide [1]. These can be either the *reintroduction* of individuals into an area formerly occupied by the species, or a *reinforcement*, which implies that individuals are released into an area already occupied by conspecifics [1–3]. Before engaging in a population restoration, several criteria must be met. The reasons causing the decline or even the extinction of the former population, for example, have to be correctly identified, removed or sufficiently reduced [1]. The area selected for the restoration must go through a habitat suitability assessment to ensure that sufficient resources are available to the target species [1].

Population restoration actions typically concern large mammals, mainly carnivores, ungulates, and primates [4,5]. Because large mammals are appealing, their reintroductions are able to attract the attention of the broader audience and indirectly favor the protection of large areas, with the potential to improve the protection of co-occurring species and habitats [6]. A growing number of reintroduction programs involve confiscated wild-born animals [7,8], which are individuals rescued from illegal pet markets or private keepers [9], mostly orphans that lost their mothers due to poaching or habitat destruction [10–12]. After confiscation, these animals can be either held in captivity for the rest of their life, returned to the wild, or euthanized [9]. Clearly, from an animal welfare perspective [13] and especially for species that are critically endangered, a return to the wild should be favored over prolonged captivity or euthanasia to improve the viability of threatened populations [5]. This also lessens the need to translocate individuals between existing wild populations for population restoration or maintaining genetic diversity.

Before being released to the wild, animals need to be *rehabilitated* to ensure they can re-adapt to wild conditions [14–16]. The length and intensity of the re-adaptation process may vary based on the history of the confiscated animals and their capability to survive on their own. At the time of confiscation some animals may have spent many years in captivity and their mental and physical state may be poor, making them suboptimal candidate founder animals [6,17]. Other individuals, however, may have spent less time in captivity as their removal from the wild occurred only recently, making them more likely to thrive upon reintroduction [18]. Candidate individuals for restoration actions must be assessed in regards to whether they possess the survival skills required for an independent life in the wild [14,16]. If necessary, they are provided with pre-release training on foraging, hunting, and predator avoidance behavior [15]. Training intensity and duration varies individually based on assessed survival capabilities, and continues until skills similar to wild conspecifics are acquired [16,19].

A prime example illustrating all the above stated challenges is the Sumatran orangutan (*Pongo abelii)*. Due to habitat loss, fragmentation, hunting and poaching for the illegal wildlife trade [11,12,20–22] the Sumatran orangutan is listed as critically endangered by the IUCN [23] with approximately 14,000 animals left in the wild [24]. Without continued habitat protection, which provides the most cost-effective long term conservation benefit [13], Sumatran orangutans are thought to be highly susceptible to extinction [25,26]. There are also at least 1500 orangutans housed in rehabilitation facilities as of 2013 [27], although this number is almost certainly an underestimate as most rehabilitation centers do not publish the number of individuals in their care. There is no decline in the number of confiscated orangutans arriving at rehabilitation facilities, the problem of illegal orangutan trade remains unsolved, and hundreds of individuals have been deployed by reintroduction programs in Sumatra and Borneo to save the species [19,28] (Pratje, personal communication). Effective orangutan conservation, therefore, also depends on the success of these releases. Survival over two dry seasons without artificial food supplementation of more than 70%, the reproduction (conception and birth) in the wild with infants being successfully reared, and a weakened link to the release sites are excellent indicators of the success of restoration actions [19]. However, the factors that influence the likelihood of such success remain generally unclear and are in need of further study.

Most confiscated orangutans are wild born and poached during infancy to accommodate the specific demand of the illegal market. The survival of these young individuals that were caught still in need of maternal care may depend on several factors, such as age at capture, individual intelligence, experience, and knowledge gained before the capture, as well as treatment received during and time spent in captivity [14,15,17]. During rehabilitation, orangutans undergo intensive training to prepare them for a life in the wild. Only basic survival skills, however, can be achieved such as physical fitness, predator avoidance behavior, ability to spend more than 95% of the time on trees without touching the ground, ability to build a nest and knowledge of a variety of common forest foods, which must make up for over one third of their total diet (Pratje, personal communication). Intensive training, however, potentially has negative side effects, such as habituation to humans, which makes it difficult to find the optimum level of training for these rehabilitation programs [15]. Immature orangutans that have not been weaned yet, for instance, strongly depend on their mother and spend most of the time close by [29]. Therefore, close contact to a caregiver during the first few years of life is very important for the development of skills such as clinging and to meet their overall psychological needs [30]. Orphaned orangutans with close contact to rehabilitation staff, however, may habituate to humans [14,15]. This effect might be stronger if the orangutans have little to no contact to other conspecifics during the rehabilitation [31]. Habituation to humans established during rehabilitation programs may influence post-release spatial behavior [14]. Habituation, however, may weaken in older individuals and with time after release: orangutans are thought to become independent over time and eventually adapt to forest life [15]. The location of the release site may additionally influence the post-release spatial behavior and prevent orangutans released far away from the release station to come back.

Very little research has been done to support or refute some of the above-mentioned speculations and to determine best strategies for rehabilitation. Empirical studies on post release behavior and habitat use are strongly needed to assess the effectiveness of rehabilitation strategies on post-release spatial behavior in the wild [1,7,32,33]. Post-release monitoring needs to assess habitat use, daily activity budgets and food selection. To date, Rijksen [34], MacKinnon [35] and Rodman [36,37] provide the most detailed and fundamental information about general behavior and habitat use of wild orangutans; Rijksen [34] includes behavioral comparisons of wild and rehabilitated orangutans, whereas spatial behavior in wild orangutans has been described by Singleton [38], Singleton & van Schaik [39] and Leighton & Leighton [40]. Direct observations can be difficult for certain species that live in remote and inaccessible locations [41]. Radio tracking, however, allows remote monitoring that can be deployed to assess post-release behavior, evaluate and improve release practices, and therefore increase survival likelihood of released individuals. Radio tracking has been deployed successfully for a variety of species, including birds [42], mammals [28], fish [43] and even insects [44]. In addition, radio tracking can reduce human contact and thus its influence on monitored wildlife species.

These factors strongly motivated our study under the umbrella of a conservation project in Sumatra, with the goal to gather information on the spatial behavior of rehabilitated and released ex-captive orangutans. Radio tracking has been commonly deployed in primates [45–51], but rarely in great apes [52].

We used radio tracking data to evaluate the post-release spatial behavior of 32 rehabilitated ex-captive Sumatran orangutans (13 females and 19 males, with age ranging from 5 to 21 years at time of release) in a population of central-eastern Sumatra, Indonesia, and compared it to the behavior expected for wild conspecifics, i.e. spatial behavior driven by food availability rather than by human presence (with special reference to people involved in the restoration program). All individuals in the study were rehabilitated orangutans which were released in an area around the Frankfurt Zoological Society (FZS) station, close to the border of the Bukit Tigapuluh National Park. The FZS station is part of the Sumatran Orangutan Conservation Project (SOCP) [53], which coordinates re-introductions of Sumatran orangutans. Our study was motivated by the urgent need to understand how these ex-captive orangutans could most successfully be prepared for a life in the wild.

We assessed how key variables—age at release, time spent in rehabilitation, time passed since the release, and the location of the release site—were correlated with features of orangutan habitat selection, specifically elevation and the distance to the nearest river—which have been identified to be strongly correlated to food availability in a previous study [54])—and distance to the rehabilitation station—where orangutans are held prior to release. The FZS station provides social contact with other orangutans and caretakers, and supplementary food provisions may be provided to returning orangutans. We modelled orangutan population-level resource selection by fitting a Resource Selection Function [55] using a Generalized Linear Model and addressed the following questions: (i) Does the orangutan age at release affect the likelihood of displaying natural spatial behavior after release, i.e. food-availability driven rather than human-presence driven? (ii) Does the duration of pre-release rehabilitation and training period affect post release spatial behavior? (iii) Do rehabilitated orangutans show signs of adjustment in spatial behavior, as expected based on the behavior of wild conspecifics, as a function of time after release? And finally (iv) does the location of the release site influence post-release spatial behavior?

## Methods

### Program and ethics information

Our study was conducted in proximity of the Frankfurt Zoological Society (FZS) station close to the border of the Bukit Tigapuluh National Park. The FZS station is part of the Sumatran Orangutan Conservation Project (SOCP) [53]. The SOCP, initiated in 1999, coordinates re-introductions of Sumatran orangutans and is based on a memorandum of understanding signed by the PanEco Foundation, the Yayasan Ekosistem Lestari (YEL), the Frankfurt Zoological Society, and the Indonesian Ministry of Forestry Directorate General of Forest Protection and Nature Conservation. The aim of the project is to provide a comprehensive conservation approach including species and ecosystem management with each partner focusing on one specific aspect. More than 360 confiscated orangutans have gone through quarantine and the related rehabilitation process so far, and more than 270 individuals have been reintroduced into the wild (Pratje, pers. communication). In 2010, the program has been evaluated and assessed as successful by the IUCN [19].The SOCP, the work of Frankfurt Zoological Society (FZS) station, as well as all procedures used in this study were approved by the Sumatran Ministry of Research, Technology and Higher Education (Kementerian Riset dan Teknologi; No.: 228/SIP/FRP/SM/VIII2014) and endorsed by the Indonesian Nature Conservation Agency (Balali Konservasi Sumber Daya Alam; LoE, 14.10.2010). All procedures also followed the IUCN guidelines for reintroduction [1,2] and major Indonesian animal welfare legislation.

### Orangutan study area

**Fig 1:**
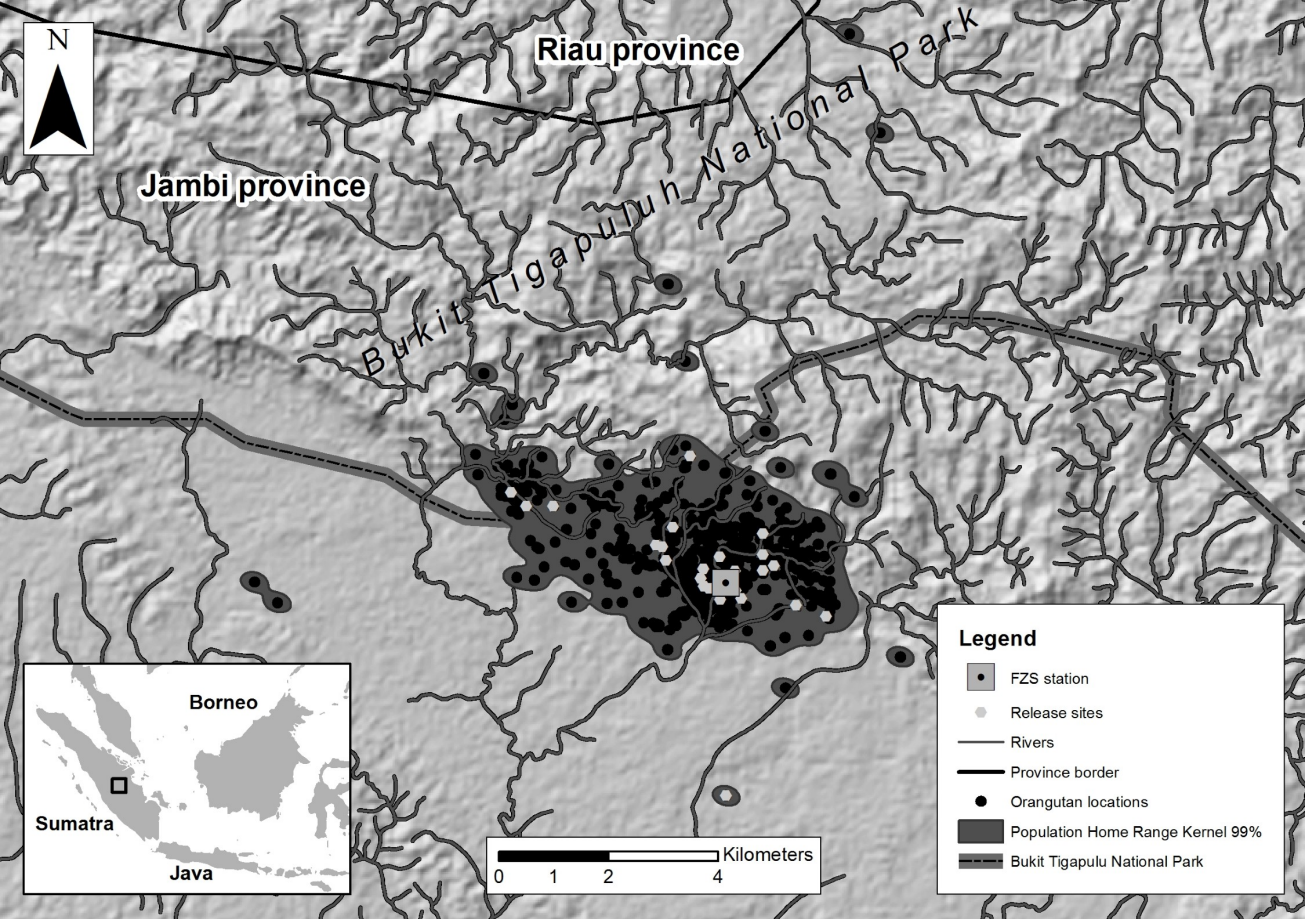
Orangutan study area located at the edge of the Bukit Tigapuluh National Park. Black dots around the Frankfurt Zoological Society (FZS) station (-1°16’N, 102°56’E) represent orangutan relocations collected using Very High Frequency radio tracking from 2011 to 2013 (n = 32 orangutans; n = 1,020 VHF relocations). Relocations were used to estimate the population-level home range (Kernel method, 99% isopleth). Grey dots represent locations where orangutans have been released. Individual orangutan maps are presented in the supporting information (S1–S8 Figs).

Our study was conducted in the 50 km^2^ area surrounding the Frankfurt Zoological Society (FZS) station, located in the Bukit Tigapuluh ecosystem of the Jambi and Riau provinces in Central Sumatra, Indonesia (Fig 1). The FZS station is located close to the border of the Bukit Tigapuluh National Park (-1°16’N, 102°56’E) and is part of the Sumatran Orangutan Conservation Program (SOCP) [15,53]. The Bukit Tigapuluh ecosystem is a lowland dipterocarp rainforest with a very rugged topography highly suitable for orangutans [56]. A dense network of small rivers and streams runs through the steep valleys. The elevation ranges between 60 and 843 meters above sea level (a.s.l.); slopes of at least 40% gradient cover more than 75% of the total area [57].

### Orangutan rehabilitation, release and radio-tracking

Since 2003, the Frankfurt Zoological Society (FZS) has released more than 160 ex-captive orangutans in this area of Sumatra following the IUCN guidelines for reintroduction [2,16]. The guidelines require that after confiscation from private households or pet markets, all orangutans have to be quarantined for a minimum of 30 days and undergo a health check, including an assessment of their physical and mental condition before entering a rehabilitation facility and having contact with other orangutans [56]. The orangutans cared for by the SOCP were transferred to the rehabilitation facility after the quarantine period. Upon arrival, individual records were created for each orangutan containing all information for identification purposes [56], such as photos of face and dentition, fingerprints, age estimate, and body measurements. Age estimates were based on dentition, bone length, body weight and behavior [58] (Ghassani, pers. communication). All orangutans had to undergo a medical check including tests for hepatitis A, B and C via blood samples and tests of fecal samples for parasites [56]. After all health checks were completed, the orangutans were transferred to socialization cages [56], where they were held with conspecifics to help them develop affiliative relationships, which sometimes continued to persist after release [14]. Socialization cages were built following IUCN guidelines: concrete floor for hygienic removal of droppings and located two meters above the ground. The cages were made of stable metal pipes with food hoist baskets and metal crates for nest building. The cages were sized approximately 8 m x 8 m x 4 m and divided into three sections, all connected by locks. Each individual went through an assessment of survival skills, such as nest-building, foraging, predator avoidance behavior and climbing skills to determine which skills were needed to be taught prior to be ready for the release [15] (Pratje, pers. communication).

During the rehabilitation process orangutans went through three main steps of the pre-release process. Firstly, they were subjected to behavioral enrichment to stimulate them mentally and physically. They practiced nest building, handling forest fruits and enhancing their motor skills, including food in hoist baskets. These baskets were provided to stimulate their interest and abilities in foraging activities by searching for food and getting it out of the object. This kind of training was performed daily. Secondly, orangutans went through forest training, where they spent the day in the forest under supervision of trainers to build up strength and practice climbing, nest building and foraging. Each orangutan underwent forest training every second day. Thirdly, orangutans had the chance to watch each other closely and develop relationships on a daily basis. This was important, since social learning plays an important role in the development of immature orangutans’ survival skills. Wild immatures normally learn through their mother [29,59], whereas rehabilitants may show the best progress when they learn from one another [15].

Orangutans were released when they showed sufficient survival capabilities. To assess survival capabilities, the orangutans were closely monitored by orangutan trainers during their forest training and in the socialization cages. In addition to being able to build a nest, foraging activity needed to reach a stable 40% of overall daily activity (Julius Paolo Siregar, personal communication). Steady movement through the trees, including the ability to move between trees and search for food independently had to reach 30% of their daily activity (Julius Paolo Siregar, personal communication). If the continuous monitoring of these individuals showed a significant increase of these capabilities for three months in a row, then an individual was deemed ready for release. This was done in the attempt to minimize the development of a strong human habituation. Table 1 reports details of the time spent in rehabilitation by the orangutans monitored in this study.

**Table 1:** Sex, estimated age at release, pre-release and release information on the 32 orangutans (OUs, 13 females, 19 males) released and monitored in central sumatra from 2011 to 2013 by means of very high frequency radio tracking. Individual spatial VHF relocations were used to build the population-level Resource Selection Function (RSF), which estimates OUs’ resource selection in a presence-available design. ‘Used’ locations (i.e., presence) correspond to sites where OUs have been relocated, whereas ‘available’ locations are random points drawn within the population home range (kernel method, 99% isopleth), which define resource availability.

OU details			Pre-release and release information		RSF’s sampling design	
Name	Sex	Estimated age at release (years)	Days of rehabilitation prior to release	Distance of the release site to the FZS station (meters)	Number of random (available) locations	Number of used (presence) locations
Suri	f	5	495	413	80	8
Miriam	f	6	701	208	70	7
Willy	f	6	417	1252	260	26
Sakdiah	f	11	138	1443	510	51
Barcelona	f	12	368	474	130	13
Chaka	f	13	114	584	480	48
Rimbani	f	13	55	164	430	43
Delavita	f	14	224	1843	70	7
Mashita	f	17	395	1526	70	7
Nathalia	f	17	168	1984	30	3
Kimong	f	21	132	806	100	10
Mutia	f	21	360	1015	340	34
Sasha	f	21	228	3527	430	43
Julius	m	5	417	1252	280	28
Ken	m	5	430	208	820	82
Jarot	m	6	168	1409	200	20
Ongki	m	6	86	222	50	5
Lindung	m	7	203	208	650	65
Mambo	m	7	701	532	480	48
Semeru	m	7	15	378	70	7
Evan	m	9	40	50	270	27
Sun_Go_Kong	m	10	336	1688	40	4
Vewe	m	10	182	575	930	93
Alex	m	12	672	556	1000	100
JunaDesky	m	12	172	1951	30	3
Nyoman	m	12	77	722	880	88
Windas	m	12	179	3937	70	7
Beckham	m	13	105	208	210	21
Joko	m	14	279	1258	310	31
Rencong	m	14	186	1764	40	4
Abel	m	16	606	3979	710	71
Mamut	m	18	194	2124	160	16
**Mean**	** **	**12 y.o.**	**276 days**	**1196 m**	**318.7**	**31.8**
**Range**		**5–21 y.o.**	**15–701 days**	**50–3979 m**	**30–1000**	**3–100**

The FZS followed a soft release regime including behavioral post release monitoring, with no feeding platforms, but supplemental food that could be provided daily within the first week after release. All animals were eventually released during the rainy season when food availability was the highest (October—March) at individual release sites that were scattered around the FZS station at different distances (Table 1). All release sites had enough resources in the surroundings, such as abundant fruit trees, water, suitable forest structure for climbing, nest-building and locomotion.

Prior to their release, between 2011 and 2013, the 32 orangutans monitored in this study (13 females, 19 males, age 5–21 years old at time of release, see Table 1) were surgically implanted with a very high frequency (VHF) transmitter, developed by the Research Institute of Wildlife Ecology, Vienna. The transmitters were implanted subcutaneously in the thick connective fat tissue of the dorsal neck area. The physical dimensions of the standard implants were 28 mm in diameter, 11 mm in height and weigh 16 g. The transmitters were made of medicinal ceramic [60] and, if the orangutan was still in proximity of the research station, were removed after two years before battery expiration (Pratje, personal communication). The transmitter emitted a VHF-signal for 8 h every day, between 07:00 and 15:00. The telemetry team performed a systematic search of radio-tagged orangutans within a 10 km radius around the release site on a weekly basis. The center of the FZS station is also the center of a path network aligned like a spider web, divided into five sectors. Once a week the projects telemetry-team followed the paths in each sector and checked for a VHF-signal every 500 meters at fixed signaling points. When an orangutan was located via triangulation, the signal was followed until the orangutan was visible [61,62] and the coordinates were determined using a GPS device (Garmin 60CSx). Over a period of 36 months the projects telemetry-team collected 1020 relocations from 32 orangutans (Table 1).

### Resource availability in relation to geographical data

We analyzed orangutan spatial behavior in relation to three different geographical datasets which have been identified as proxies for resource availability in a previous study at the same study site [54]: the location of the FZS station, the digital elevation model, and the location of rivers.

The FZS station provided released orangutans with social contact to other orangutans waiting to be released and with staff involved in the pre-release training and other activities. The FZS station provided supplementary fruit provisions for returning orangutans which appeared to be undernourished or rejected by wild orangutans. Note that orangutans were not released at the FZS station, but at individual release sites scattered at varying distances around the station (see Fig 1, S1–S8 Figs and below for full details).

Elevation was a good proxy for food availability since the number and abundance of fruit tree species increased with decreasing elevation, with the highest abundance being around 150–250 m a.s.l. [54]. Raster information on elevation (digital elevation model) was derived from the NASA Shuttle Radar Topographic Mission (SRTM) at 90 m resolution and was resampled to a spatial resolution of 30 m using regularized splines with tension interpolation [63].

The distance to the nearest river was another proxy for food availability since the highest number and abundance of fruit tree species were located in riparian forest areas close to rivers [54]. The most prominent family of fruit trees was *Moraceae*, which has short and frequent fructification cycles [64,65], thus providing a year-round accessible stable food resource [54]. Locations of the rivers in our study area were provided by the Indonesian Forestry Ministry.

### Orangutan population-level resource selection

We modeled orangutan population-level resource selection by fitting a Resource Selection Function (RSF) [55]. A RSF is defined as any statistical model deployed to estimate the relative probability of selecting a resource unit versus alternative possible resource units. Resource selection functions are particularly suited to presence-availability designs, where used resources are sampled at locations where animals are relocated (presence data), whereas resources sampled at random locations within the area the animals could potentially use are used to characterize resource availability [55]. In our study case we followed Manly’s design II [55], meaning that the resources used by monitored orangutans were sampled at the individual level—i.e., radio tracking relocations—whereas available resources were sampled at the population level—i.e. random points within the population home range. Population level home range was calculated using the Kernel method (99% isopleth) [66]. Manly’s design II is recommended when the number of relocations for some of the monitored individuals is low, i.e., when the computation of availability at the individual home range level (e.g. Manly’s design III) is not suitable because of insufficient number of points needed to define it. A sensitivity analysis showed that at least seven random points associated to each orangutan relocation were needed to obtain stable model estimates. Therefore, we opted to draw ten random points per used location to obtain robust parameter estimates [67] (Table 1).

For each ‘used’ and ‘available’ orangutan location, we computed the distance to the FZS station, the distance to the nearest river and the elevation using ArcGIS 10.2 [68]. Each “used” location and related available random locations were associated with orangutan name, age, sex, the time spent in rehabilitation (in days), the time after release (in days), and the distance of the release site to the FZS station (Table 1). We thus created a dataset with ‘used’ (1s) and ‘available’ (0s) as a binary response variable. Predictors were screened to exclude collinearity issues (Pearson correlation coefficient |*r*_*p*_| < 0.7; variance inflation factor VIF < 3; [69,70]).

A RSF approach implies two main steps. First, we need to estimate selection coefficients (i.e., beta estimates) using a logistic model. Second, we have to plug the coefficients estimated by the logistic model into a resource selection function [71–73]. We thus fitted a Generalized Linear Model (GLM) with a binomial distribution of error (step 1). We hypothesized the selection for the environmental predictors—i.e., distance to the FZS station, distance to river, elevation, including quadratic terms to account for nonlinear effects—to vary depending on orangutan age, time spent in rehabilitation, and time after release. We thus included these interaction terms in our starting model. To test whether animals released closer to the FZS station were more bonded to it, we added the interaction term between the distance to the FZS station and the distance of the release site to the FZS station. We finally included orangutan sex and orangutan identity (name) as fixed factors in the model to account for sex differences in habitat selection and for pseudoreplication of data, respectively. We chose to run a GLM with orangutan name as fixed effect rather than a mixed effect model (GLMM: [69,74]) with orangutan identity as random intercept. Both model classes properly account for pseudoreplication; however, because we could not meet the main assumption of mixed models, i.e., normally distributed random intercepts, we opted for the more robust GLM. We eventually simplified the structure of our starting GLM using the *stepAIC* function of the MASS package in R [75] to find the most parsimonious model based on the Akaike Information Criterium [76].

Parameters estimated of the best GLM were used to depict RSF predictions for orangutan selection patterns (step 2). The RSF was assumed to take the exponential form [55] as follows:

$$ŵ(x)=exp(\beta_{1}x_{1}+\beta_{2}x_{2}+\beta_{3}x_{3}+\ldots +\beta_{n}x_{n})$$ (Eq 1)

where β_1_ to β_n_ are coefficients estimated by the GLM, which are associated with environmental variables x_1_ to x_n_, respectively. All statistical analyses were performed in R version 3.2.3 [75].

## Results

**Fig 2:**
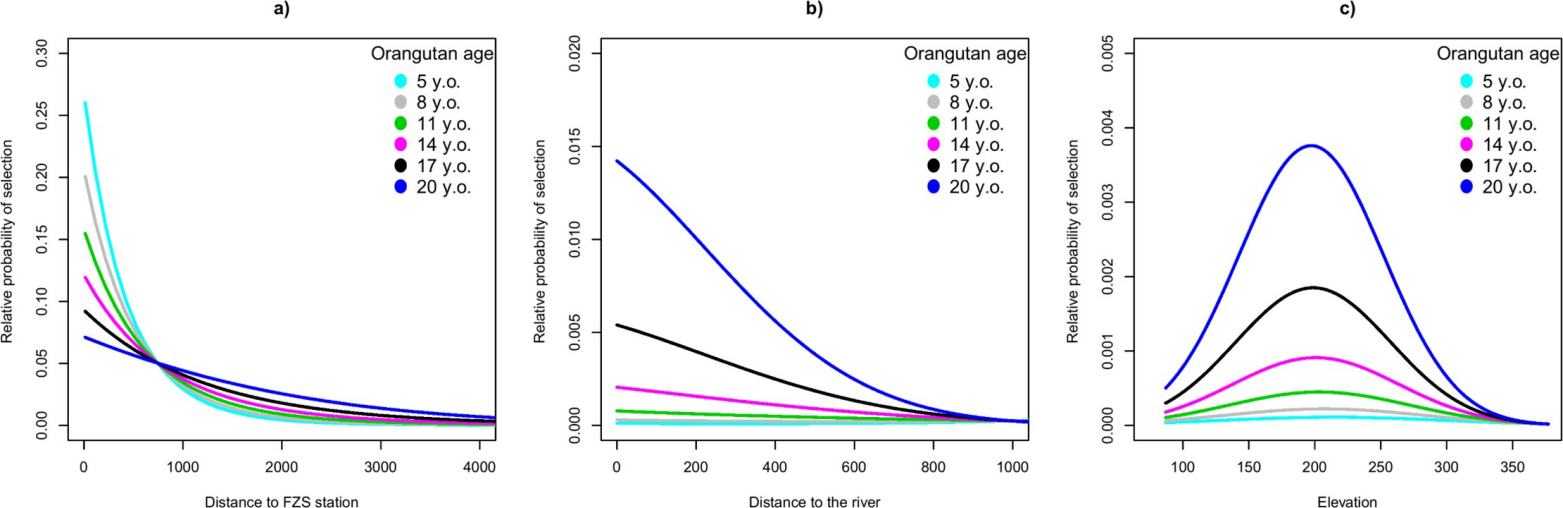
Habitat selection patterns become more pronounced in older orangutans. Relative probability of selection in 32 orangutans (n = 13 females, n = 19 males) as a function of orangutan age (six different scenarios for age, in y.o.) interacted with the distance to the FZS station (a, in meters), the distance to the nearest river (b, in meters), and elevation (c, in meters). The lines represent the average relative probability of selection as predicted by the resource selection function.

**Fig 3:**
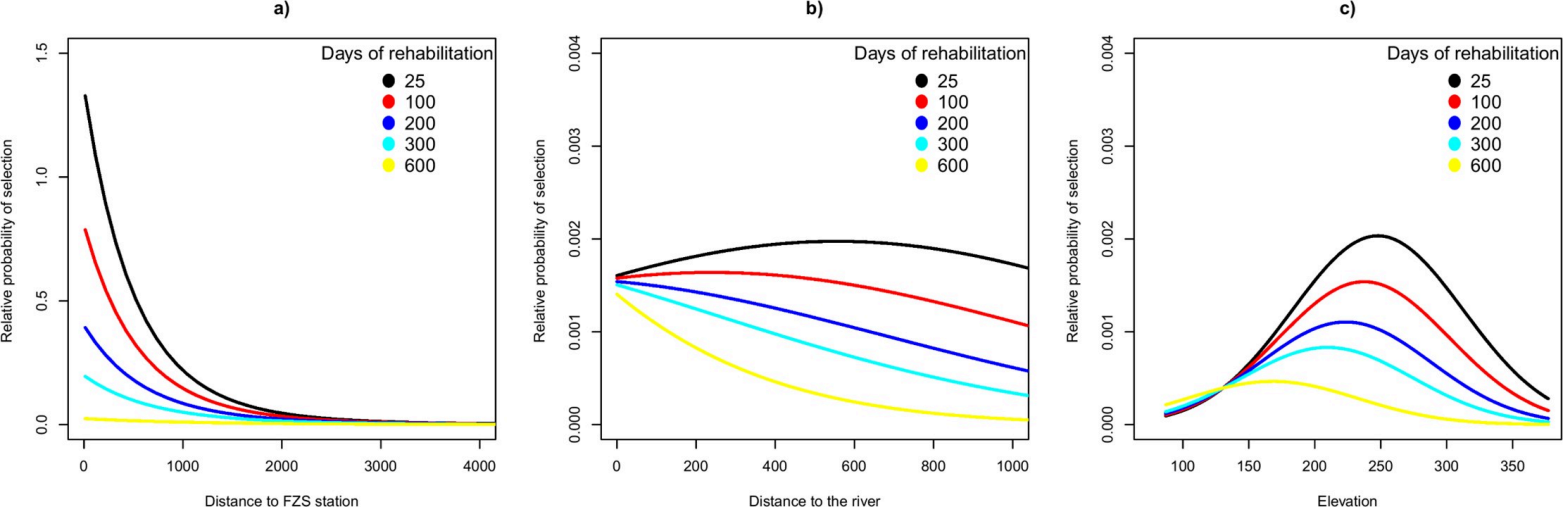
Time spent in rehabilitation affects resource selection patterns. Relative probability of selection in 32 orangutans (n = 13 females, n = 19 males) as a function of the number of days spent in rehabilitation prior to their release (five different scenarios) interacted with the distance to the FZS station (a, in meters), the distance to the nearest river (b, in meters), and elevation (c, in meters). The lines represent the average relative probability of selection as predicted by the resource selection function.

**Fig 4:**
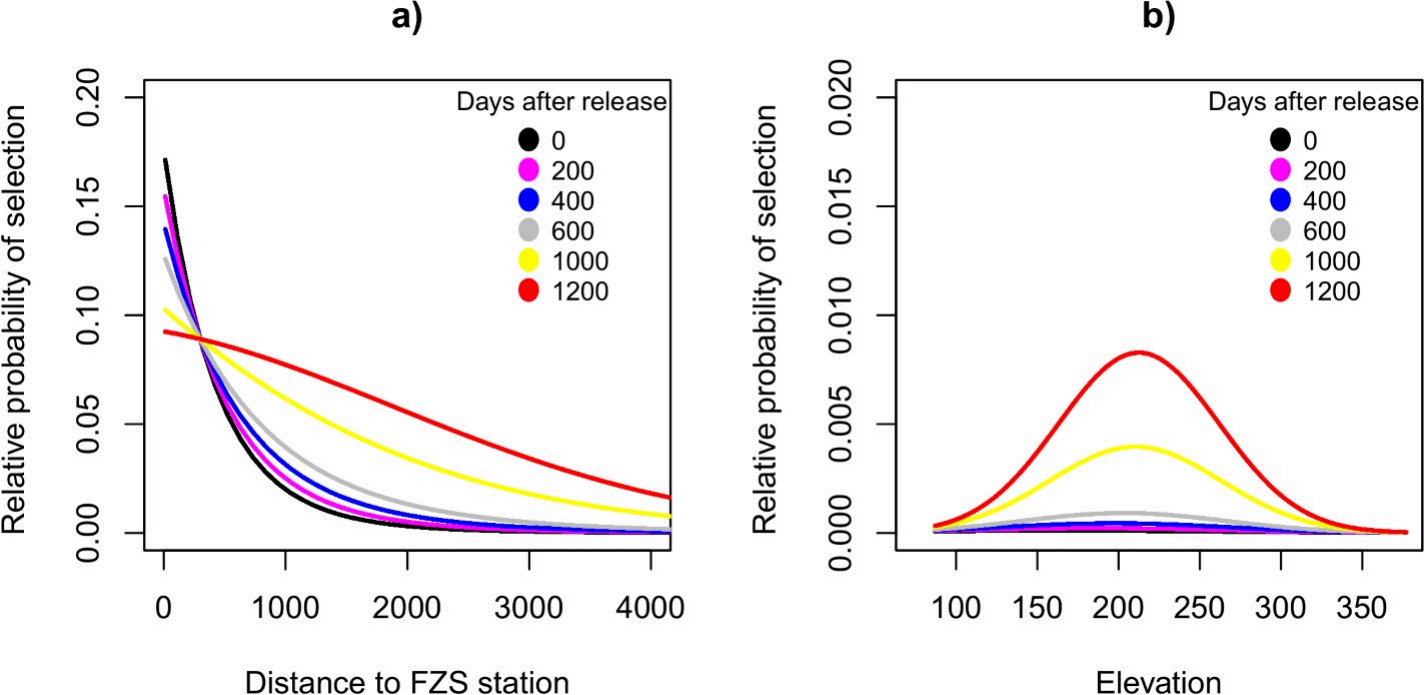
Orangutans adjust resource selection over time after release. Relative probability of selection in 32 orangutans (n = 13 females, n = 19 males) as a function of time after release (six different scenarios, in days) interacted with the distance to the FZS station (a, in meters), and elevation (b, in meters). The lines represent the average relative probability of selection as predicted by the resource selection function.

**Fig 5:**
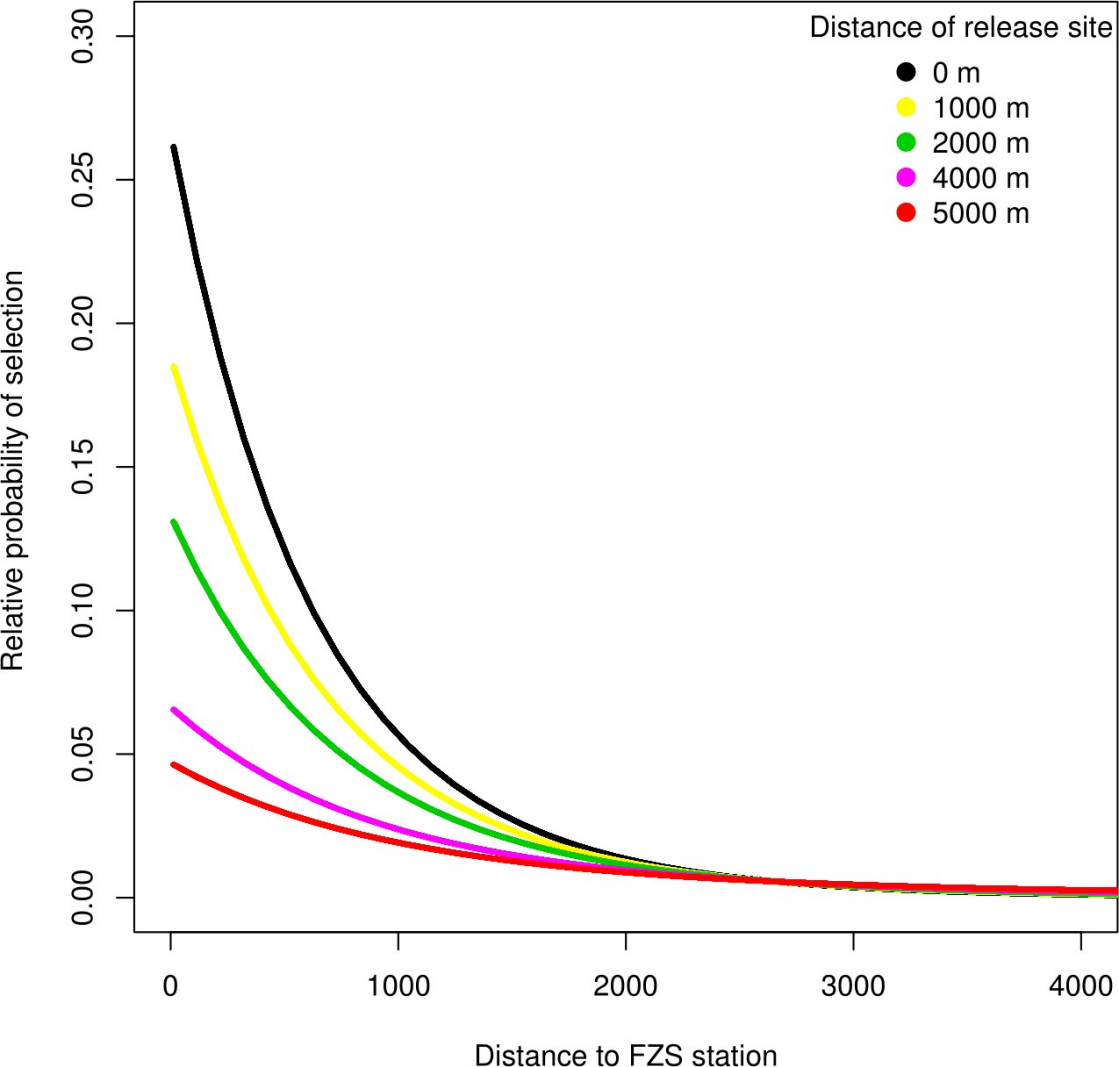
Individuals released further from the FZS station show less inclination to spend time in its vicinity. Relative probability of selection in 32 orangutans (n = 13 females, n = 19 males) as a function of the distance of the release site from the FZS station (five different scenarios, in meters) interacted with the distance to the FZS station (in meters). The lines represent the average relative probability of selection as predicted by the resource selection function.

The step AIC model selection procedure allowed us to simplify the structure of the starting full GLM (AIC = 3333.4), which resulted in the final version reported in Table 2 (AIC = 3323.7, pseudo-R^2^ = 0.53). The final model, explaining more than 50% of the variance in habitat selection and post-release spatial behavior in rehabilitated orangutans, retained most of the interaction terms; the sex of orangutans, however, was not retained (Table 2). The parameters estimated by the best GLM were plugged in the RSF (Eq 1) to depict orangutan selection patterns: interaction terms were all significant and were portrayed in Figs 2–5 (for detailed model predictions depicting inter-individual variability as well as model uncertainty see supplementary information, S9–S13 Figs).

**Table 2:** Parameters estimated by the Generalized Linear Model (GLM) fitted to depict population-level resource selection by orangutans from 2011 to 2013 in Central Sumatra.

Model coefficients	β estimate	Standard Error	Z value	P value	
intercept	2.934000	1.646000	1.782	0.075	
elevation	-0.003822	0.017030	-0.224	0.822	
elevation^2^	0.000036	0.000045	0.807	0.419	
distance to the FZS station	-0.004932	0.000329	-14.985	<0.001	***
distance to the FZS station^2^	4.171E-07	3.916E-08	10.652	<0.001	***
distance to the river	0.000888	0.000771	1.152	0.250	
distance to the river^2^	0.000004	0.000001	3.698	<0.001	***
distance of the release site to the FZS station	-0.000348	0.000195	-1.786	0.074	
age	-0.206500	0.130600	-1.582	0.114	
age × elevation	0.002481	0.001263	1.965	0.049	*
age × elevation^2^	-0.000007	0.000003	-1.954	0.051	
age × distance to the FZS station	0.000128	0.000022	5.906	<0.001	***
age × distance to the FZS station^2^	-1.319E-08	2.748E-09	-4.799	<0.001	***
age × distance to the river^2^	-3.251E-07	8.682E-08	-3.745	<0.001	***
days of rehabilitation	0.003806	0.001437	2.649	0.008	**
days of rehabilitation x elevation	-0.000033	0.000005	-6.254	<0.001	***
days of rehabilitation x distance to the FZS station	1.837E-06	4.458E-07	4.119	<0.001	***
days of rehabilitation x distance to the FZS station^2^	-2.205E-10	6.608E-11	-3.337	<0.001	***
days of rehabilitation x distance to the river	-0.000006	0.000001	-4.517	<0.001	***
days after release	-0.007220	0.002352	-3.069	0.002	**
days after release × elevation	0.000059	0.000026	2.273	0.023	*
days after release × elevation^2^	-1.289E-07	6.859E-08	-1.879	0.060	
days after release × distance to the FZS station	0.000002	4.275E-07	4.358	<0.001	***
days after release × distance to the FZS station^2^	-2.017E-10	6.086E-11	-3.315	<0.001	***
distance to the FZS station × distance of the release site to the FZS station	1.312E-07	3.450E-08	3.803	<0.001	***

Beta coefficients estimated by the GLM were eventually plugged in the Resource Selection Function (RSF)—which we assumed to take the exponential form—resulting in the resource selection patterns depicted in Figs 2–5. Sample size (used and available locations): n = 11,528 relocations for 32 orangutans (13 females and 19 males). Pseudo-R^2^: 0.53

Significant codes

* p ≤ 0.05

** p ≤ 0.01

*** p ≤ 0.001

Reference orangutan of parameter estimation: Abel. Intercept estimates for the other 31 OUs (*i*.*e*., inter-individual variability): mean β = -0.05792 (range: -1.258–1.53); mean SE = 0.582 (range 0.300–0.965).

We found that the relative probability of selection for the distance to the FZS station, the distance to the nearest river, and elevation significantly varied with orangutan age (significant interaction between age and environmental predictors, Table 2 and Fig 2A–2C). Younger orangutans selected for areas closer to the FZS station (Fig 2A). Older individuals, in contrast, showed a stronger selection for areas closer to rivers (Fig 2B) and for areas located at an elevation expected to be optimal for food availability (~200 m a.s.l., Fig 2C). Age of monitored orangutans ranged between 5 and 21 years old. The scenarios depicted in Fig 2 are meant to show the development of resource selection patterns in our tracked orangutans as a function of their age, from younger to older ones. See supplementary information for detailed model predictions (S9 and S10 Figs) and for a comprehensive overview of the shift in resource selection patterns in increasingly older monitored individuals.

We found that the time spent in rehabilitation affected resource selection patterns (significant interaction between time spent in rehabilitation and environmental predictors, Table 2 and Fig 3). Orangutans that spent more time in rehabilitation were found to be less likely closer to the FZS station (Fig 3A), more likely to be closer to rivers (Fig 3B), and selected for areas located at lower elevations (where food availability is expected to be higher), even though the latter pattern was weaker (Fig 3C). See supplementary information (S11 Fig) for detailed predictions depicting inter-individual variability and model uncertainty.

We found that the resource selection by the 32 orangutans varied as a function of the time after release (significant interaction between time after release and environmental predictors, Table 2 and Fig 4). Orangutans significantly decreased the selection for areas closer to the FZS station over time after their release (Fig 4A), as well as they increased the selection for elevations around 200 meters a.s.l. (Fig 4B, see Supplementary information S12 Fig for detailed predicted patterns).

Finally, we found that orangutans that were released far from the FZS station were less likely to be bonded to it (significant interaction between distance to the FZS station and the distance of the release site to the FZS station, Table 2 and Fig 5; supplementary information S13 Fig).

## Discussion

We made use of radio tracking data to determine how key variables drove the post-release spatial behavior and habitat selection of rehabilitated orangutans in central-eastern Sumatra, Indonesia. We showed that the age at release, the duration of pre-release rehabilitation training, the time since release and the location of the release site positively affected the likelihood of released orangutans to assume food-availability-driven spatial behavior in the wild.

### Age at release plays a key role in post-release spatial behavior

We found that younger orangutans were more likely to range closer to the FZS station, while older individuals were generally more likely to exhibit spatial behavior similar to wild counterparts. The strong spatial bond to the FZS station in younger orangutans was most likely related to the need for social contacts and food [14,77,78]. Given that young wild orangutans still strongly depend on their mother and spend most of the time in close proximity [29], it is plausible that the FZS station and the people there are assuming this function. Although extended effort was put into teaching the orangutans the needed survival skills, e.g. nest building, foraging, predator avoidance behavior and climbing, they still have a disadvantage to their wild conspecifics. The spatial bond to the FZS station decreased with increasing age at release. This is in accordance with the natural emancipation process, which implies that orangutans become independent from their mother and try to establish a home range of their own [29]. Previous research showed that orangutans that stayed at greater distance from the release station displayed spatial behavior that was more similar to that shown by wild conspecifics of similar age [15]. Although actual behavior of the orangutans was not measured, the recorded spatial behavior in this study indicates a similar pattern (see below for further discussion). In contrast, orangutans remaining in the vicinity of the release station may still be underdeveloped [15,79,80].

Whereas habitat selection in younger orangutans was mainly driven by their attraction to the FZS station, older orangutans showed strong selection for areas closer to the rivers with riparian forest. In addition to the highest number and abundance of fruit tree species [54], these areas have the highest frequency of large-diameter trees, and provide the most suitable forest structure for locomotion [81], indicating it is the best suitable habitat. With increasing age, selection for elevation around 200 m a.s.l. also became more pronounced. Compared to the maximum elevation in our study area (843 m a.s.l.), areas located at 200 m a.s.l. are expected to be inhabited by several fruit tree species and provide abundant fruit availability [54]. However, these patterns may be also related to older orangutans becoming increasingly dominant [82,83] and thus possibly occupying the better suitable habitat, forcing the younger orangutans to move to less suitable areas. The current release practice favors releasing orangutans at a young age, i.e. as juveniles or adolescents, because this stage of semi-independence is believed to be the best period for learning and integration [14,84]. Based on our findings, however, we suggest releasing orangutans at an older age.

### Duration of pre-release rehabilitation and training affects post-release spatial behavior

We found that orangutans benefit from longer pre-release rehabilitation and training. Most ex-captive orangutans have limited capabilities to survive on their own [18]. Therefore, it is imperative to utilize their high learning abilities [14,29,85,86] and provide sufficient pre-release training [14,16]. The benefits of longer training are a higher chance to acquire needed survival skills, including the possibility to learn from more experienced conspecifics while held in socializing cages [14,16,87]. Therefore, the SOCP rehabilitation program has had to walk a fine line between providing sufficient pre-release training and releasing orangutans as soon as possible to minimize the chance of habituation. Social contact and care of apes by humans creates strong affiliation to humans [78], which is believed to have a negative influence on the adaptation process of rehabilitated orangutans [15]. In contrast to that, our results showed that the orangutans with longer pre-release training showed stronger selection for areas with higher food availability and a reduced bond to the release station, mimicking behavior expected from wild conspecifics. This indicates that pre-release training outweighs the possible negative effects of the developments of strong affiliations to humans.

### Spatial behavior changes with increasing time after release

Our data showed a significant change in habitat selection over time after release. Other primate reintroduction and release programs report that the greatest behavioral change happens in the first year after release [88]. Irrespective of age, with increasing time after release orangutans in our study showed selection for habitat at greater distance from the FZS station and their selection for elevation became more pronounced. This is especially important for young orangutans. A recent study of the same population [89] found that survival rates of young rehabilitated orangutans are lower than those of young wild conspecifics [25,90], which is most likely related to immature foraging skills, narrowly based habitat selection (this study), and poor predator avoidance behavior [15]. The increasing distance to the release station and the more pronounced selection for elevation with increasing time after release is evidence of adaptation to more natural ranging behavior [15,89]. With more time after release, orangutans become more experienced and confident [29], and start exploring areas further away from the release station, thus eventually finding other areas with stable food supplies.

### Location of the release site influences post-release spatial behavior

Our data suggest that orangutans released at greater distances from the FZS station were less likely to go back to it. Therefore, if habitat suitability is ensured [16], releasing orangutans as far away as possible from human presence (in this case the FZS station), might be desirable to foster emancipation. However, it is possible that geographical barriers and the difficulty traversing the terrain contribute to the finding that orangutans released near the FZS station were more likely to stay in its vicinity. Also, orangutans released near to the release station have a greater chance of being in hearing distance and therefore might be drawn back to it. With increasing distance of the release site from the FZS station new challenges arise, including logistics and increased transportation stress for the orangutans [16]. In addition, the inaccessibility might lead to low-intensity post release monitoring. Therefore, only orangutans with a very high level of survival skills should be released far away from the release station. A closely monitored release near the release station might be preferable for less experienced orangutans.

### Limitations of this study

The unknown pre-confiscation history of the rehabilitated orangutans remains a challenge in explaining individual differences in behavior and abilities during pre- and post-release. Depending on the age at capture the orangutans may or may not have learned basic survival skills from their mothers [18,80]. For most orangutans the treatment during captivity is unknown [11]. Some orangutans are held as pets and might have developed a strong affiliation to humans [15], others were abused during captivity and might have developed an aversion against humans, which might influence their post-release spatial behavior. In long-term projects such as release programs for orangutans, data continuity is a big problem and data quality often suffers from constant staff rotation. To prevent data loss standardized data sheets should be developed and used, e.g. for the initial health assessment or individual daily records during the rehabilitation process. Projects with geographically separated facilities for quarantine, rehabilitation and release should also have standardized hand-over procedures and documentation for all relevant data. Only then we will be able to increase our fundamental understanding of future behavioral development of rehabilitated orangutans.

## Conclusions

The variables we selected (i.e. age at release, the duration of pre-release rehabilitation training, the time since release and the location of the release site) explained more than fifty percent of the variance in habitat selection and post release spatial behavior in rehabilitated orangutans. However, other factors, such as semi-solitary lifestyle [91], age-dependent avoidance behavior [34,83] or the relatively high orangutan density in close proximity to release stations [54] should be taken into account in future studies in order to deepen our understanding of post-release spatial behavior in rehabilitated orangutans.

It has been argued that the best age to be released for orangutans is as juveniles or adolescents, because this stage of semi-independence is believed to be the best period for learning and integration [14,84]. However, this is in contrast with our findings, and it motivates further research to shed lights on all factors affecting the success of reintroduction and release programs. Based on our findings, we suggest favoring extended training over a fast release. Orangutans with a high level of survival skills should be released at greater distances to foster emancipation from humans. Orangutans with improvable survival skills, which have developed a bond with another, more experienced orangutan during rehabilitation should be released in pairs to facilitate learning [15,18]. Finally, we advocate continued daily monitoring for all released orangutans, so that habitat selection and movement patterns can be observed and better understood. To achieve this goal, research and development of Global Positioning Systems (GPS) transmitters suitable for orangutans and other great apes living an arboreal lifestyle is of great importance.

## Supporting information

S1 Fig **Individual maps for female orangutans Suri (5 years old at time of release, 8 relocations, top left), Miriam (6 years old at time of release, 7 relocations, top right), Willy (6 years old at time of release, 26 relocations, bottom left) and Sakdiah (11 years old at time of release, 51 relocations, bottom right).** Red dots represent locations where orangutans have been released. Blue dots represent orangutan relocations. Green lines represent individual orangutan home range boundaries (MCP 100%). Blue lines represent orangutan population home range boundaries (Kernel 99%). Grey square with black dot represents location of the FZS station.
(TIF)

S2 Fig **Individual maps for female orangutans Barcelona (12 years old at time of release, 13 relocations, top left), Chaka (13 years old at time of release, 48 relocations, top right), Rimbani (13 years old at time of release, 43 relocations, bottom left) and Delavita (14 years old at time of release, 7 relocations, bottom right).** Red dots represent locations where orangutans have been released. Blue dots represent orangutan relocations. Green lines represent individual orangutan home range boundaries (MCP 100%). Blue lines represent orangutan population home range boundaries (Kernel 99%). Grey square with black dot represents location of the FZS station.
(TIF)

S3 Fig **Individual maps for female orangutans Mashita (17 years old at time of release, 7 relocations, top left), Nathalia (17 years old at time of release, 3 relocations, top right), Kimong (21 years old at time of release, 10 relocations, bottom left) and Mutia (21 years old at time of release, 34 relocations, bottom right).** Red dots represent locations where orangutans have been released. Blue dots represent orangutan relocations. Green lines represent individual orangutan home range boundaries (MCP 100%). Blue lines represent orangutan population home range boundaries (Kernel 99%). Grey square with black dot represents location of the FZS station.
(TIF)

S4 Fig **Individual map for female orangutan Sasha (21 years old at time of release, 43 relocations, top left) and male orangutans Julius (5 years old at time of release, 28 relocations, top right), Ken (5 years old at time of release, 82 relocations, bottom left) and Jarot (6 years old at time of release, 20 relocations, bottom right).** Red dots represent locations where orangutans have been released. Blue dots represent orangutan relocations. Green lines represent individual orangutan home range boundaries (MCP 100%). Blue lines represent orangutan population home range boundaries (Kernel 99%). Grey square with black dot represents location of the FZS station.
(TIF)

S5 Fig **Individual maps for male orangutans Ongki (6 years old at time of release, 5 relocations, top left), Lindung (7 years old at time of release, 65 relocations, top right), Mambo (7 years old at time of release, 48 relocations, bottom left) and Semeru (7 years old at time of release, 7 relocations, bottom right)**. Red dots represent locations where orangutans have been released. Blue dots represent orangutan relocations. Green lines represent individual orangutan home range boundaries (MCP 100%). Blue lines represent orangutan population home range boundaries (Kernel 99%). Grey square with black dot represents location of the FZS station.
(TIF)

S6 Fig **Individual maps for male orangutans Evan (9 years old at time of release, 27 relocations, top left), Sun_Go_Kong (10 years old at time of release, 4 relocations, top right), Vewe (10 years old at time of release, 93 relocations, bottom left) and Alex (12 years old at time of release, 100 relocations, bottom right).** Red dots represent locations where orangutans have been released. Blue dots represent orangutan relocations. Green lines represent individual orangutan home range boundaries (MCP 100%). Blue lines represent orangutan population home range boundaries (Kernel 99%). Grey square with black dot represents location of the FZS station.
(TIF)

S7 Fig **Individual maps for male orangutans JunaDesky (12 years old at time of release, 3 relocations, top left), Nyoman (12 years old at time of release, 88 relocations, top right), Windas (12 years old at time of release, 7 relocations, bottom left) and Beckham (13 years old at time of release, 21 relocations, bottom right)**. Red dots represent locations where orangutans have been released. Blue dots represent orangutan relocations. Green lines represent individual orangutan home range boundaries (MCP 100%). Blue lines represent orangutan population home range boundaries (Kernel 99%). Grey square with black dot represents location of the FZS station.
(TIF)

S8 Fig **Individual maps for male orangutans Joko (14 years old at time of release, 31 relocations, top left), Rencong (14 years old at time of release, 4 relocations, top right), Abel (16 years old at time of release, 71 relocations, bottom left) and Mamut (18 years old at time of release, 16 relocations, bottom right)**. Red dots represent locations where orangutans have been released. Blue dots represent orangutan relocations. Green lines represent individual orangutan home range boundaries (MCP 100%). Blue lines represent orangutan population home range boundaries (Kernel 99%). Grey square with black dot represents location of the FZS station.
(TIF)

S9 Fig Relative probability of selection in 32 orangutans (n = 13 females, n = 19 males) as a function of orangutan age (six different scenarios for age, in years old–but see S10 Fig for a full overview of age scenarios) interacted with the distance to the FZS station (first row, in meters), the distance to the nearest river (second row, in meters), and elevation (third row, in meters). The black line represents the average value, other lines represent the parameter uncertainty related to inter-individual variability as predicted by the resource selection function.
(PDF)

S10 Fig Relative probability of selection in 32 orangutans (n = 13 females, n = 19 males) as a function of orangutan age interacted with the distance to the FZS station (first row, in meters), the distance to the nearest river (second row, in meters), and elevation (third row, in meters). The black line represents the average value; other lines represent the parameter uncertainty related to inter-individual variability as predicted by the resource selection function. Age of monitored orangutans ranged from 5 to 21 years old, here depicted by nine age scenarios representing the evolution of resource selection patterns from younger to older individuals of the sample size.
(TIFF)

S11 Fig Relative probability of selection in 32 orangutans (n = 13 females, n = 19 males) as a function of the number of days spent in rehabilitation prior to their release (seven different scenarios) interacted with the distance to the FZS station (first row, in meters), the distance to the nearest river (second row, in meters), and elevation (third row, in meters). The black line represents the average value, other lines represent the parameter uncertainty related to inter-individual variability as predicted by the resource selection function.
(PDF)

S12 Fig Relative probability of selection in 32 orangutans (n = 13 females, n = 19 males) as a function of time after release (seven different scenarios, in days) interacted with the distance to the FZS station (first row, in meters), and elevation (second row, in meters). The black line represents the average value, other lines represent the parameter uncertainty related to inter-individual variability as predicted by the resource selection function.
(PDF)

S13 Fig Relative probability of selection in 32 orangutans (n = 13 females, n = 19 males) as a function of the distance of the release site from the FZS station (six different scenarios, in meters) interacted with the distance to the FZS station (in meters). The black line represents the average value, other lines represent the parameter uncertainty related to inter-individual variability as predicted by the resource selection function.
(PDF)

S1 File OUdatabase. **VHF orangutan relocations used in the analysis.** Note that GPS coordinates were censored to avoid potential poachers to visit the areas used by monitored individuals.
(TXT)

## References

[1] IUCN/SSC. IUCN guidelines for reintroductions and other conservation translocations. Ecologial Applications. Gland, Switzerland; 2013. 10.1016/j.biocon.2015.07.030

[2] IUCN. Guidelines for re-introduction. Gland, Switzerland and Cambridge, UK; 1998.

[3] Soorae PS, Seddon PJ. Re-introduction practitioners directory. Gland, Switzerland: IUCN Species Survival Commission Reintroduction Specialist Group; 1998.

[4] Seddon PJ, Soorae PS, Launay F. Taxonomic bias in reintroduction projects. Anim Conserv. 2005;8: 51–58. 10.1017/S1367943004001799

[5] Guy AJ, Curnoe D, Banks PB. Welfare based primate rehabilitation as a potential conservation strategy: does it measure up? Primates. 2014;55: 139–47. 10.1007/s10329-013-0386-y 24132600

[6] Seddon PJ, Armstrong DP, Maloney RF. Developing the science of reintroduction biology. Conserv Biol. 2007;21: 303–12. 10.1111/j.1523-1739.2006.00627.x 17391180

[7] Cheyne SM. Wildlife reintroduction: considerations of habitat quality at the release site. BMC Ecol. 2006;6: 5 10.1186/1472-6785-6-5 16611369 PMC1458321

[8] Guy AJ, Curnoe D, Banks PB. A survey of current mammal rehabilitation and release practices. Biodivers Conserv. 2013;22: 825–837. 10.1007/s10531-013-0452-1

[9] IUCN/SSC Re-introduction specialist group, Environmental research & wildlife development agency (Emirats Arabes Unis). IUCN guidelines for the placement of confiscated animals. Gland, Switzerland: IUCN; 2002.

[10] Cowlishaw G, Dunbar RIM. Primate Conservation Biology. Chicago: University of Chicago Press; 2000.

[11] Rijksen HD, Meijaard E. Our Vanishing Relative: The Status of Wild Orang-utans at the Close of the Twentieth Century. Dordrecht: Kluwer Academic Publishers; 1999.

[12] Singleton I, Wich SA, Hosson S, Stephens S, Utami-Atmoko SS, Leighton M, et al Orangutan population and habitat viability assessment: Final report. Apple Valley, MN; 2004.

[13] Wilson HB, Meijaard E, Venter O, Ancrenaz M, Possingham HP. Conservation Strategies for Orangutans: Reintroduction versus Habitat Preservation and the Benefits of Sustainably Logged Forest. Ryan SJ, editor. PLoS One. Public Library of Science; 2014;9: e102174 10.1371/journal.pone.0102174 25025134 PMC4099073

[14] Russon AE. Orangutan rehabilitation and reintroduction In: Wich SA, Atmoko SSU, Seita T, Van Schaik CP, editors. Orangutans Geographic Variations in Behavioral Ecology and Conservation. Oxford: Oxford University Press; 2009 pp. 327–350.

[15] Riedler B, Millesi E, Pratje PH. Adaptation to forest life during the reintroduction process of immature Pongo abelii. Int J Primatol. 2010;31: 647–663. 10.1007/s10764-010-9418-2

[16] Beck B, Walkup K, Rodrigues M, Unwin S, Travis D, Stoinski T. Best practice guidelines for the re-introduction of great apes. Gland, Switzerland: SSC Primate Specialist Group of the World Conservation Union; 2007.

[17] Baker LR. Guidelines for nonhuman primate re-introductions. Re-introduction NEWS. 2002;21: 29–57.

[18] Russon AE. Return of the Native: Cognition and Site-Specific Expertise in Orangutan Rehabilitation. Int J Primatol. 2002;23: 461–478. 10.1023/A:1014909431148

[19] Soorae PS. Global re-introduction perspectives: Additional case studies from around the globe. Abu Dhabi, UAE: IUCN, SSC Re-introduction Specialist Group; 2010.

[20] Wich SA, Meijaard E, Marshall AJ, Husson S, Ancrenaz M, Lacy RC, et al Distribution and conservation status of the orang-utan (Pongo spp.) on Borneo and Sumatra: how many remain? Oryx. 2008;42: 329–339. 10.1017/S003060530800197X

[21] Meijaard E, Buchori D, Hadiprakarsa Y, Utami-Atmoko SS, Nurcahyo A, Tjiu A, et al Quantifying killing of orangutans and human-orangutan conflict in Kalimantan, Indonesia. PLoS One. 2011;6: e27491 10.1371/journal.pone.0027491 22096582 PMC3214049

[22] Stiles D, Redmond I, Cress D, Nellemann C, Formo RK. Stolen Apes* The Illicit Trade in Chimpanzees, Gorillas, Bonobos, and Orangutans In: Haenn N, Wilk RR, Harnish A, editors. The Environment in Anthropology: A Reader in Ecology, Culture, and Sustainable Living. Secon edit New York: New York University Press; 2016 p. 359.

[23] IUCN. The IUCN Red List of Threatened Species. Version 2017.3. 2017.

[24] Wich SA, Singleton I, Nowak MG, Utami Atmoko SS, Nisam G, Arif SM, et al Land-cover changes predict steep declines for the Sumatran orangutan (*Pongo abelii*). Sci Adv. 2016;2. 10.1126/sciadv.1500789 PMC4783118 26973868

[25] Ellis S, Singleton I, Andayani N, Traylor-Holzer K, Supriatna J. Sumatran orangutan conservation action plan. Ellis S, Singleton I, Andayani N, Traylor-Holzer K, Supriatna J, editors. Washington D.C.; Jakarta Indonesia: Conservation International; 2006.

[26] Marshall AJ, Lacy R, Ancrenaz M, Byers O, Husson SJ, Leighton M, et al Orangutan population biology, life history, and conservation In: Wich SA, Atmoko SSU, Seita TM, Van Schaik CP, editors. Orangutans Geographic Variations in Behavioral Ecology and Conservation. Oxford: Oxford University Press; 2009 pp. 311–326.

[27] Trayford HR, Farmer KH. Putting the Spotlight on Internally Displaced Animals (IDAs): A Survey of Primate Sanctuaries in Africa, Asia, and the Americas. Am J Primatol. Wiley-Blackwell; 2013;75: 116–134. 10.1002/ajp.22090 23097324

[28] Soorae PS. Global Re-introduction Perspectives, 2013: Further Case Studies from Around the Globe. Gland, Switzerland: IUCN/SSC Re-introduction Specialist Group & Environment Agency-Abu Dhabi; 2013.

[29] van Noordwijk MA, Sauren SEB, Nuzuar AA, Morrogh-Bernard HC, Van Schaik CP. Development of independance. Sumatran and Bornean orangutans compared In: Wich SA, Atmoko SSU, Seita TM, Van Schaik CP, editors. Orangutans Geographic Variations in Behavioral Ecology and Conservation. Oxford: Oxford University Press; 2009 pp. 189–203.

[30] Sodaro C. Orangutan Species Survival Plan Husbandry Manual. Chicago, IL: Chicago Zoological Park; 1997.

[31] Fritz J, Fritz P. The hand-rearing unit: Management decisions that may affect chimpanzee development Clin Manag infant Gt apes. Alan R Liss Inc, New York; 1985; 1–34.

[32] Armstrong DP, Seddon PJ. Directions in reintroduction biology. Trends Ecol Evol. 2008;23: 20–5. 10.1016/j.tree.2007.10.003 18160175

[33] King T, Chamberlan C, Courage A. Assessing initial reintroduction success in long-lived primates by quantifying survival, reproduction, and dispersal parameters: Western Lowland Gorillas (Gorilla gorilla gorilla) in Congo and Gabon. Int J Primatol. 2011;33: 134–149. 10.1007/s10764-011-9563-2

[34] Rijksen HD. A field study on Sumatran orang utans (*Pongo pygmaeus abelii* Lesson 1827): ecology, behaviour and conservation. Wageningen. 1978.

[35] MacKinnon J. The behaviour and ecology of wild orang-utans (*Pongo pygmaeus*). Anim Behav. Elsevier; 1974;22: 3–74.

[36] Rodman P. Feeding behaviour of orang-utans of the Kutai Nature Reserve, East Kalimantan In: CluttonBrock TH, editor. Primate Ecology: studies of feeding and ranging behaviour in lemurs, monkeys and apes. London: Academic Press; 1977 pp. 384–414.

[37] Rodman P. Diversity and consistency in ecology and behavior. In: Schwarz J, editor. The Orangutan. Oxford: Oxford University Press New York; 1988 pp. 31–51.

[38] Singleton I. Ranging behaviour and seasonal movements of Sumatran orangutans (*Pongo pygmaeus abelii*) in swamp forests. University of Kent 2000.

[39] Singleton I, van Schaik CP. Orangutan Home Range Size and Its Determinants in a Sumatran Swamp Forest. Int J Primatol. 2001;22: 877–911. 10.1023/A:1012033919441

[40] Leighton M, Leighton D. Vertebrate responses to fruiting seasonality within a Bornean rain forest In: Sutton SL, Withmore TC, Chadwick AC, editors. Tropical Rain Forest: Ecology and Management. Boston: Blackwell Scientific Pub; 1983 pp. 181–196.

[41] Markham AC, Altmann J. Remote monitoring of primates using automated GPS technology in open habitats. Am J Primatol. 2008;70 10.1002/ajp.20515 18176947

[42] Bernardo CSS, Lloyd H, Olmos F, Cancian LF, Galetti M. Using post‐release monitoring data to optimize avian reintroduction programs: a 2‐year case study from the Brazilian Atlantic Rainforest. Anim Conserv. Wiley Online Library; 2011;14: 676–686.

[43] Jellyman D. A review of radio and acoustic telemetry studies of freshwater fish in New Zealand. Mar Freshw Res. CSIRO; 2009;60: 321–327.

[44] Kissling DW, Pattemore DE, Hagen M. Challenges and prospects in the telemetry of insects. Biol Rev. Wiley/Blackwell (10.1111); 2014;89: 511–530. 10.1111/brv.12065 24106908

[45] Qi X-G, Garber PA, Ji W, Huang Z-P, Huang K, Zhang P, et al Satellite telemetry and social modeling offer new insights into the origin of primate multilevel societies. Nat Commun. Nature Publishing Group; 2014;5: 5296 10.1038/ncomms6296 25335993 PMC4220467

[46] Guy AJ, Stone OML, Curnoe D. Assessment of the release of rehabilitated vervet monkeys into the Ntendeka Wilderness Area, KwaZulu-Natal, South Africa: A case study. Primates. 2012; 10.1007/s10329-011-0292-0 22258755

[47] Dias C, Queirogas V, Pedersoli M. Translocation and radio-telemetry monitoring of pygmy marmoset, *Cebuella pygmaea* (Spix, 1823), in the Brazilian Amazon. Brazilian J Biol. 2015; 10.1590/1519-6984.07813 25945625

[48] Fedigan LM, Fedigan L, Chapman C, Glander KE. Spider monkey home ranges: A comparison of radio telemetry and direct observation. Am J Primatol. Wiley Subscription Services, Inc., A Wiley Company; 1988;16: 19–29. 10.1002/ajp.1350160104 31968876

[49] Juarez CP, Rotundo MA, Berg W, Fernández-Duque E. Costs and benefits of radio-collaring on the behavior, demography, and conservation of Owl Monkeys (*Aotus azarai*) in Formosa, Argentina. Int J Primatol. Springer US; 2011;32: 69–82. 10.1007/s10764-010-9437-z

[50] Brown M, Crofoot M. Social and spatial relationships between primate groups In: Sterling E, Bynum N, Blair M, editors. Primate Ecology and Conservation. Oxford: Oxford University Press; 2013 pp. 151–176. 10.1093/acprof:oso/9780199659449.003.0009

[51] Klegarth AR. Measuring movement: how remote telemetry facilitates our understanding of the human–macaque interface In: Dore KM, Riley EP, Fuentes A, editors. Ethnoprimatology: A Practical Guide to Research at the Human-Nonhuman Primate Interface. Cambridge: Cambridge University Press; 2017 pp. 70–87.

[52] Nayasilana IKEN, Hadisusanto S, Wijayanto H, Atmoko SRISU, Prasetyo D, Sihite J, et al Behavioral ecology of reintroduced Orangutans in the Bukit Batikap, Central Kalimantan, Indonesia. Biodiversitas J Biol Divers. 2017;18: 875–886.

[53] Sumatran Orangutan Conservation Project [Internet]. [cited 23 Aug 2018]. Available: https://www.sumatranorangutan.org/

[54] Kelle D, Gärtner S, Pratje PH, Storch I. Reintroduced Sumatran Orangutans (Pongo abelii): using major food tree species as indicators of habitat suitability. Folia Primatol (Basel). 2014;85: 90–108. 10.1159/000357498 24504132

[55] Manly BFJ, McDonald LL, Thomas DL, McDonald TL, Erickson WP. Resource Selection by Animals: Statistical Analysis and Design for Field Studies, 2nd Edition Nordrecht: Kluwer Academic Publishers; 2002.

[56] Trayford H, Pratje P, Singleton I. Reintroduction of the sumatran orangutan in Sumatra, Indonesia In: Soorae PS, editor. Global Re-introduction Perspectives: Additional case-studies from around the globe. Abu Dhabi, UAE: IUCN, SSC Re-introduction Specialist Group; 2010 pp. 238–242.

[57] Whitten T, Damanik SJ, Anwar J, Hisyam N. The Ecology of Sumatra. Hong Kong: Periplus Editions; 1997.

[58] Kuze N, Dellatore D, Banes GL, Pratje P, Tajima T, Russon AE. Factors affecting reproduction in rehabilitant female orangutans: young age at first birth and short inter-birth interval. Primates. 2012;53: 181–92. 10.1007/s10329-011-0285-z 22109351

[59] van Noordwijk MA, van Schaik CP. Development of ecological competence in Sumatran orangutans. Am J Phys Anthropol. 2005;127: 79–94. 10.1002/ajpa.10426 15472890

[60] Walzer C, Petit T, Nathan S, Boklin C, Sipangkui S, Fluch G. Small VHF-implants for radio tracking reintroduced freeranging orangutans (*Pongo pygmaeus*). Abstract 2010 proceedings AAZV AAWV Joint conference. 2010.

[61] Kenward R. A Manual for Wildlife Radio Tagging, 2nd edition San Diego: Academic Press; 2001.

[62] Cooke S. Biotelemetry and biologging in endangered species research and animal conservation: relevance to regional, national, and IUCN Red List threat assessments. Endanger Species Res. 2008;4: 165–185. 10.3354/esr00063

[63] Mitášová H, Mitáš L. Interpolation by regularized spline with tension: I. Theory and implementation. Math Geol. 1993;25: 641–655. 10.1007/BF00893171

[64] van Schaik CP. Phenological changes in a Sumatran rain forest. J Trop Ecol. Cambridge Univ Press; 1986;2: 327–347. 10.1017/S0266467400000973

[65] Wich SA, Utami-Atmoko SS, Setia TM, Djoyosudharmo S, Geurts ML. Dietary and energetic responses of *Pongo abelii* to fruit availability fluctuations. Int J Primatol. Springer; 2006;27: 1535–1550. 10.1007/s10764-006-9093-5

[66] Worton BJ. Kernel methods for estimating the utilization distribution in home-range studies. Ecology. Eco Soc America; 1989;70: 164–168. 10.2307/1938423

[67] Ciuti S, Tripke H, Antkowiak P, Gonzalez RS, Dormann CF, Heurich M. An efficient method to exploit LiDAR data in animal ecology. Warton D, editor. Methods Ecol Evol. Wiley/Blackwell (10.1111); 2018;9: 893–904. 10.1111/2041-210X.12921

[68] ESRI. ArcGIS. Redlands, CA: ESRI; 2014.

[69] Zuur A, Ieno EN, Walker N, Saveliev AA, Smith GM. Mixed Effects Models and Extensions in Ecology with R. New York: Springer; 2009.

[70] Dormann CF, Elith J, Bacher S, Buchmann C, Carl G, Carré G, et al Collinearity: a review of methods to deal with it and a simulation study evaluating their performance. Ecography (Cop). Wiley/Blackwell (10.1111); 2013;36: 27–46. 10.1111/j.1600-0587.2012.07348.x

[71] Manly BF, McDonald LL, Thomas DL, McDonald TL, Erickson WP. Resource Selection by Animals: Statistical Design and Analysis for Field Studies. Dordrecht, The Netherlands: Kluwer Academic Publishers; 2002.

[72] Thurfjell H, Ciuti S, Boyce MS. Applications of step-selection functions in ecology and conservation. Mov Ecol. 2014;2: 4 10.1186/2051-3933-2-4 25520815 PMC4267544

[73] Boyce MS, Vernier PR, Nielsen SE, Schmiegelow FKA. Evaluating resource selection functions. Ecol Model. 2002;157 10.1016/S0304-3800(02)00200-4

[74] Pinheiro JC, Bates DM. Mixed-Effects Models in S and S-PLUS. New York: Springer-Verlag; 2000 10.1007/b98882

[75] R Core Team. R: A language and environment for statistical computing. Vienna, Austria: R Foundation for Statistical Computing; 2015.

[76] Akaike H. Likelihood of a model and information criteria. J Econom. North-Holland; 1981;16: 3–14.

[77] Yeager CP. Orangutan rehabilitation in Tanjung Puting National Park, Indonesia. Conserv Biol. 1997;11: 802–805. 10.1046/j.1523-1739.1997.95500.x

[78] Russon AE, van Schaik CP, Kuncoro P, Ferisa A, Handayani DP, Van Noordwijk MA. Innovation and intelligence in orangutans In: Wich SA, Atmoko SSU, Seita T, van Schaik CP, editors. Orangutans: Geographic variation in behavioral ecology and conservation. Oxford: Oxford University Press; 2009 pp. 279–298.

[79] Russon AE. Developmental perspectives on great ape traditions In: Fragaszy DM, Perry S, editors. The Biology of Traditions: Models and Evidence. Cambridge: Cambridge University Press; 2003 pp. 329–364.

[80] Russon AE, Handayani DP, Kuncoro P, Ferisa A. Orangutan leaf-carrying for nest-building: toward unraveling cultural processes. Anim Cogn. 2007;10: 189–202. 10.1007/s10071-006-0058-z 17160669

[81] Manduell KL, Harrison ME, Thorpe SKS. Forest structure and support availability influence orangutan locomotion in Sumatra and Borneo. Am J Primatol. 2012;74: 1128–1142. 10.1002/ajp.22072 22915011

[82] Singleton I, van Schaik CP. The social organisation of a population of Sumatran orang-utans. Folia Primatol. Karger Publishers; 2002;73: 1–20. 10.1159/000060415 12065937

[83] Utami-Atmoko SS, Singleton I, van Noordwijk MA, van Schaik CP, Setia TM. Male-male relationships in orangutans In: Wich SA, Utami-Atmoko SS, Setia TM, van Schaik CP, editors. Orangutans, Geographic Variations in Behavioral Ecology and Conservation. Oxford: Oxford University Press; 2009 pp. 225–233.

[84] Rosen N, Byers O. Orangutan conservation and reintroduction workshop: final report. Apple Valley, MN: IUCN/SSC Conservation Breeding Specialist Group; 2002.

[85] Cocks L, Bullo K. The progress for releasing a zoo-bred Sumatran orang-utan (*Pongo abelii*) at Bukit Tigapuluh National Park, Jambi, Sumatra. Int Zoo Yearb. 2008;42: 1–7.

[86] Grundmann E, Lestel D, Boestani AN, Bomsel MC. Learning to survive in the forest: What every orangutan should know. The Apes: Challenges for the 21st Century, Conference Proceedings. Chicago, IL: Brookfield Zoo; 2000. pp. 300–304.

[87] van Schaik CP. The socioecology of fission-fusion sociality in orangutans. Primates. Springer; 1999;40: 69–86. 10.1007/BF02557703 23179533

[88] Stoinski TS, Beck BB. Changes in locomotor and foraging skills in captive born, reintroduced golden lion tamarins (*Leontopithecus rosalia rosalia*). Am J Primatol. 2004;62: 1–13. 10.1002/ajp.20002 14752809

[89] Kelle D, Fechter D, Singer A, Pratje P, Storch I. Determining sensitive parameters for the population viability of reintroduced sumatran orangutans (Pongo abelii). Int J Primatol. 2013;34: 423–442. 10.1007/s10764-013-9671-2

[90] Wich SA, Utami-Atmoko SS, Setia TM, Rijksen HD, Schürmann C, van Hooff JARAM, et al Life history of wild Sumatran orangutans (*Pongo abelii*). J Hum Evol. 2004;47: 385–98. 10.1016/j.jhevol.2004.08.006 15566945

[91] Delgado RA, Van Schaik CP. The behavioral ecology and conservation of the orangutan (*Pongo pygmaeus*): A tale of two islands. Evol Anthropol Issues, News, Rev. 2000;9: 201–218. 10.1002/1520-6505(2000)9:5

