# Supplementary figures and images for: Spatial behavior in rehabilitated orangutans in Sumatra: Where do they go?
Source: PLoS One. 2019 May 1;14(5):e0215284. doi: 10.1371/journal.pone.0215284 (PMC6493737; doi:10.1371/journal.pone.0215284)

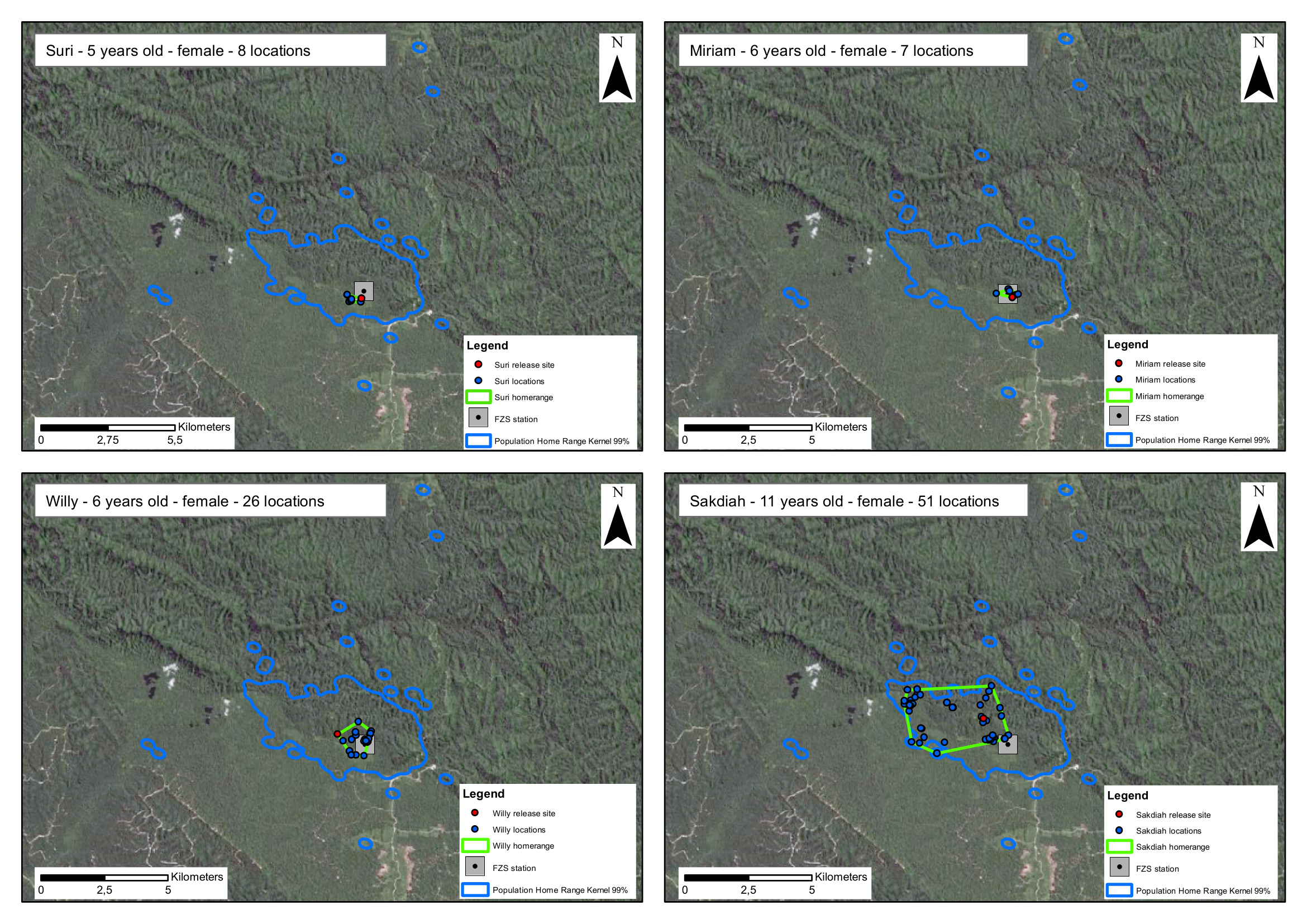

Supplement: S1 Fig — Individual maps for female orangutans Suri (5 years old at time of release, 8 relocations, top left), Miriam (6 years old at time of release, 7 relocations, top right), Willy (6 years old at time of release, 26 relocations, bottom left) and Sakdiah (11 years old at time of release, 51 relocations, bottom right). Red dots represent locations where orangutans have been released. Blue dots represent orangutan relocations. Green lines represent individual orangutan home range boundaries (MCP 100%). Blue lines represent orangutan population home range boundaries (Kernel 99%). Grey square with black dot represents location of the FZS station. (TIF) [file pone.0215284.s001.tif]

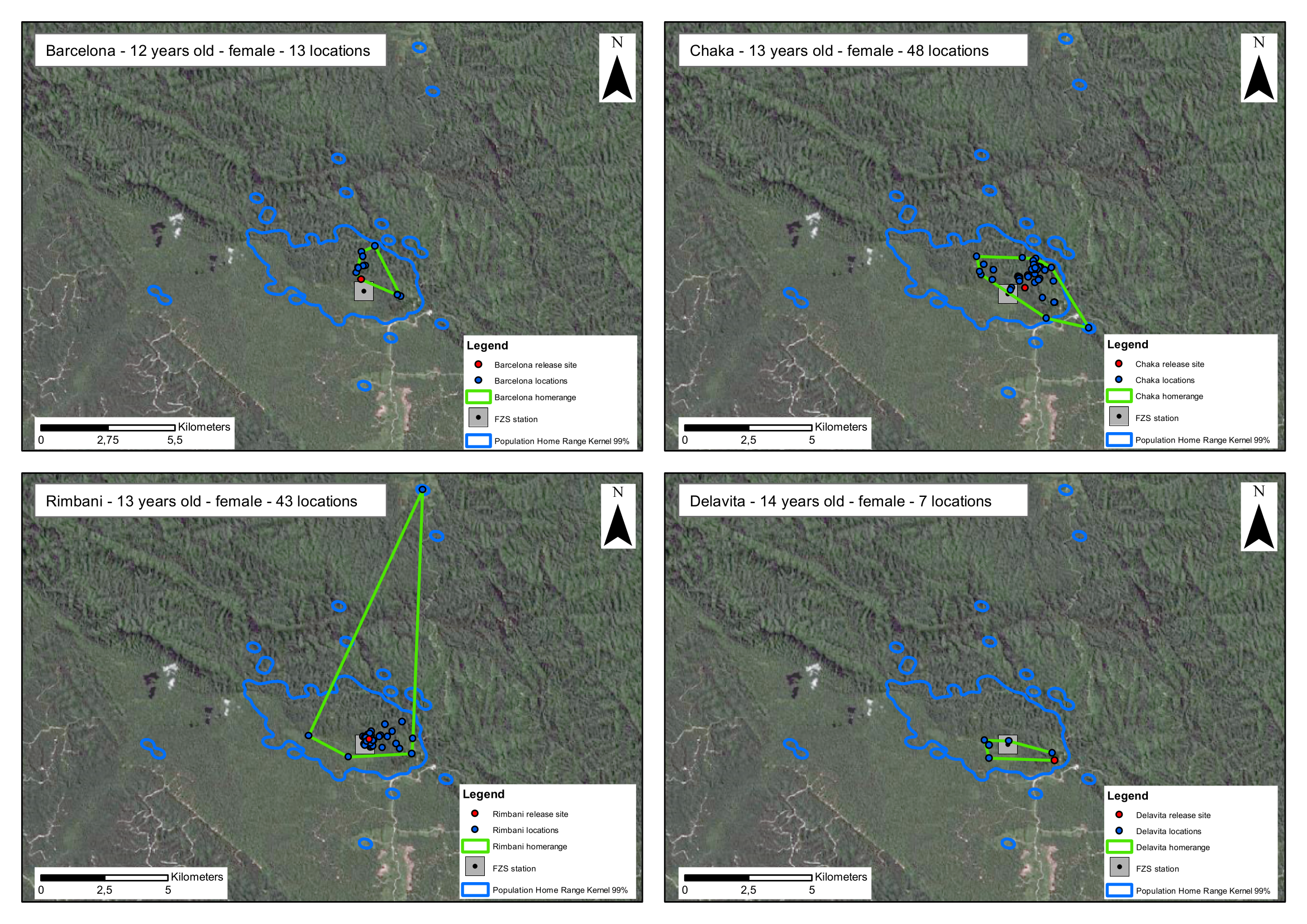

Supplement: S2 Fig — Individual maps for female orangutans Barcelona (12 years old at time of release, 13 relocations, top left), Chaka (13 years old at time of release, 48 relocations, top right), Rimbani (13 years old at time of release, 43 relocations, bottom left) and Delavita (14 years old at time of release, 7 relocations, bottom right). Red dots represent locations where orangutans have been released. Blue dots represent orangutan relocations. Green lines represent individual orangutan home range boundaries (MCP 100%). Blue lines represent orangutan population home range boundaries (Kernel 99%). Grey square with black dot represents location of the FZS station. (TIF) [file pone.0215284.s002.tif]

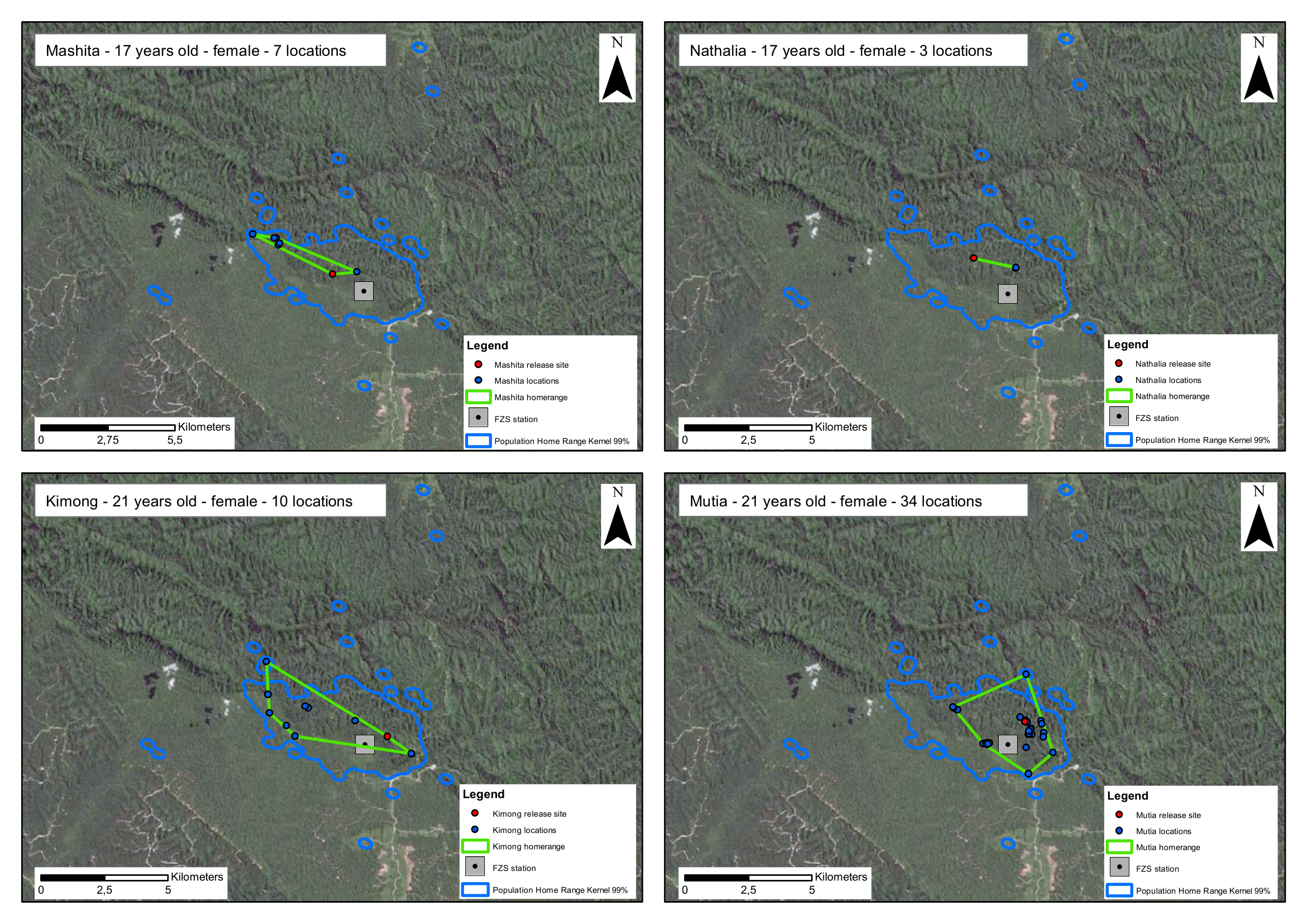

Supplement: S3 Fig — Individual maps for female orangutans Mashita (17 years old at time of release, 7 relocations, top left), Nathalia (17 years old at time of release, 3 relocations, top right), Kimong (21 years old at time of release, 10 relocations, bottom left) and Mutia (21 years old at time of release, 34 relocations, bottom right). Red dots represent locations where orangutans have been released. Blue dots represent orangutan relocations. Green lines represent individual orangutan home range boundaries (MCP 100%). Blue lines represent orangutan population home range boundaries (Kernel 99%). Grey square with black dot represents location of the FZS station. (TIF) [file pone.0215284.s003.tif]

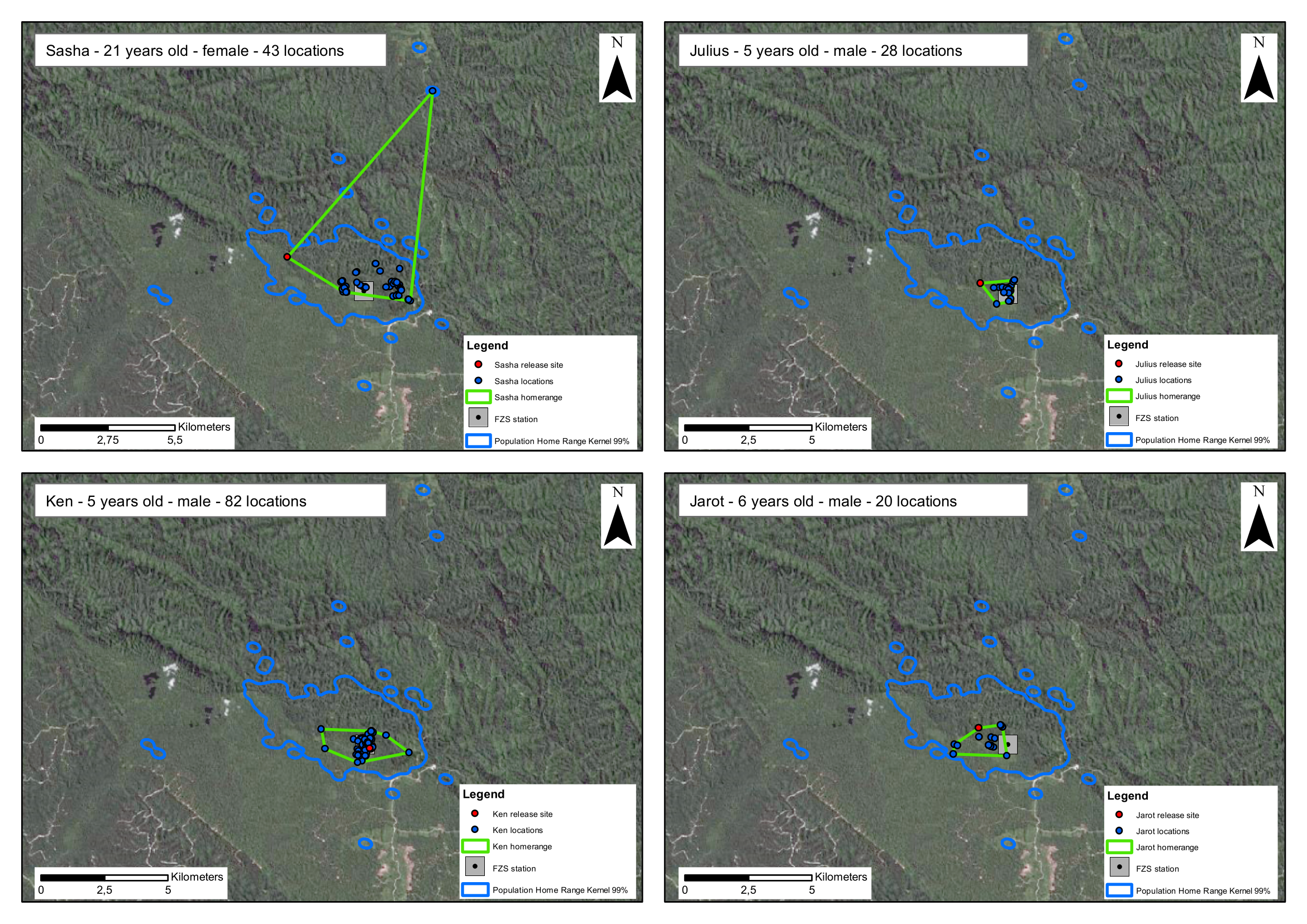

Supplement: S4 Fig — Individual map for female orangutan Sasha (21 years old at time of release, 43 relocations, top left) and male orangutans Julius (5 years old at time of release, 28 relocations, top right), Ken (5 years old at time of release, 82 relocations, bottom left) and Jarot (6 years old at time of release, 20 relocations, bottom right). Red dots represent locations where orangutans have been released. Blue dots represent orangutan relocations. Green lines represent individual orangutan home range boundaries (MCP 100%). Blue lines represent orangutan population home range boundaries (Kernel 99%). Grey square with black dot represents location of the FZS station. (TIF) [file pone.0215284.s004.tif]

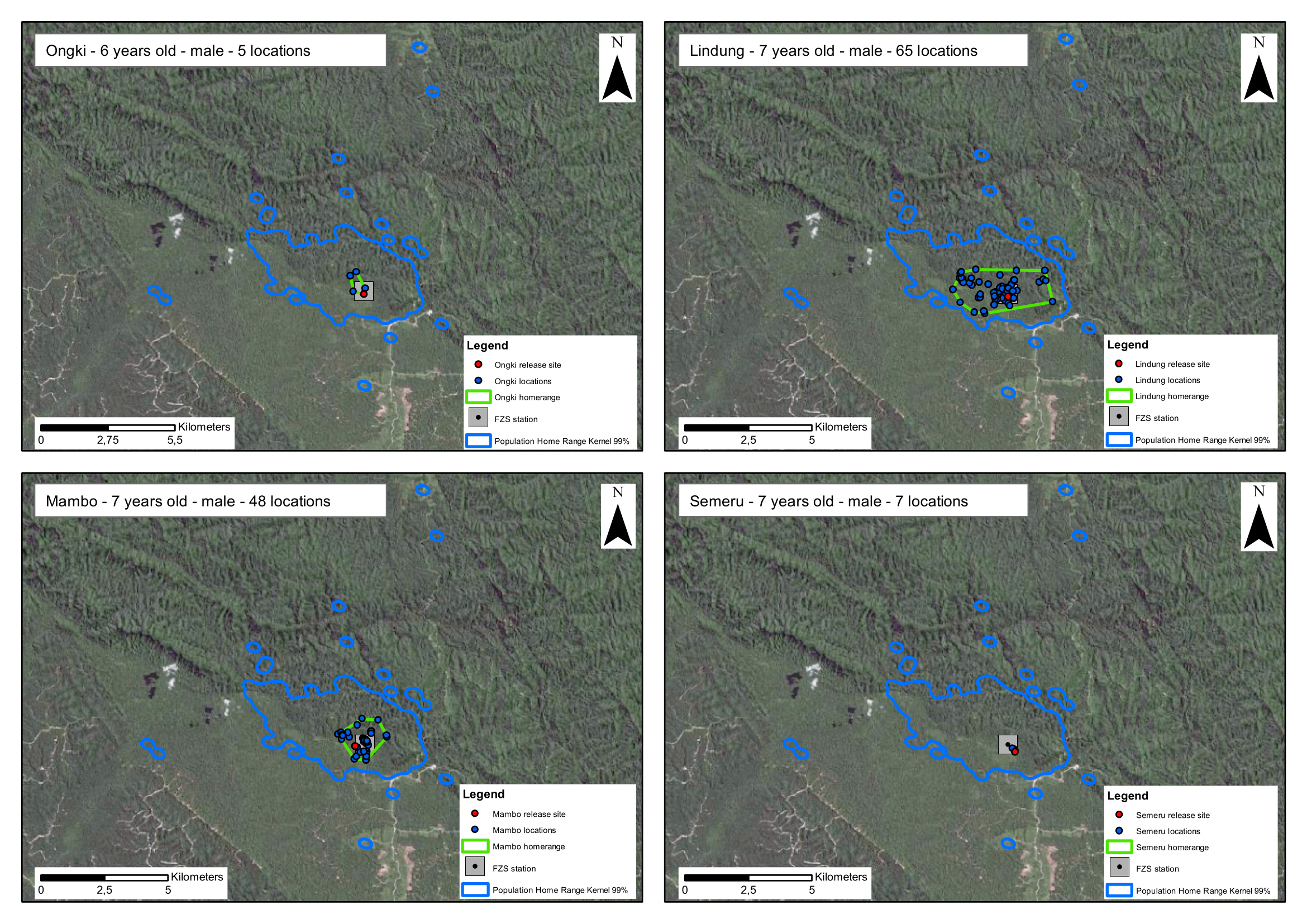

Supplement: S5 Fig — Individual maps for male orangutans Ongki (6 years old at time of release, 5 relocations, top left), Lindung (7 years old at time of release, 65 relocations, top right), Mambo (7 years old at time of release, 48 relocations, bottom left) and Semeru (7 years old at time of release, 7 relocations, bottom right). Red dots represent locations where orangutans have been released. Blue dots represent orangutan relocations. Green lines represent individual orangutan home range boundaries (MCP 100%). Blue lines represent orangutan population home range boundaries (Kernel 99%). Grey square with black dot represents location of the FZS station. (TIF) [file pone.0215284.s005.tif]

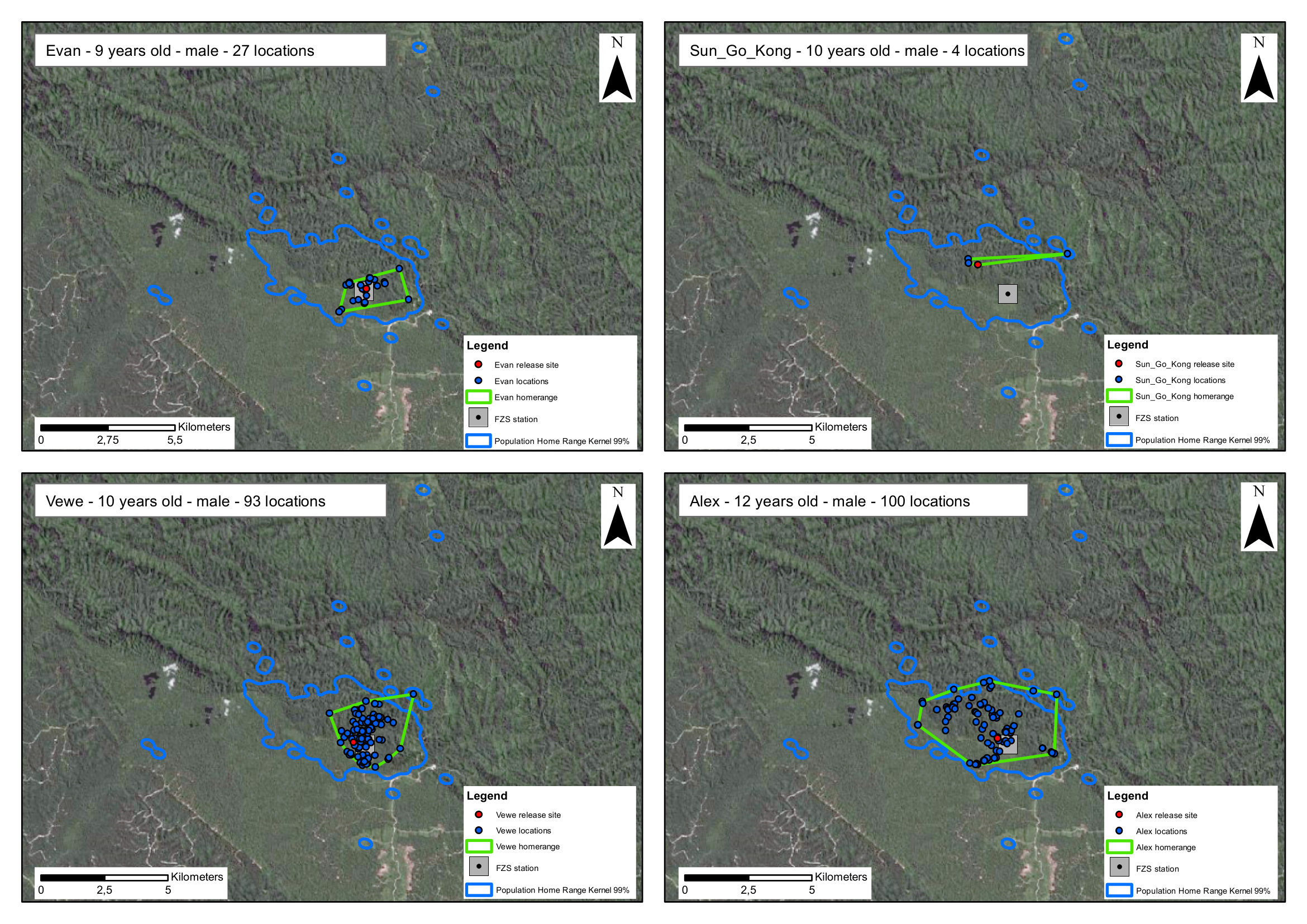

Supplement: S6 Fig — Individual maps for male orangutans Evan (9 years old at time of release, 27 relocations, top left), Sun_Go_Kong (10 years old at time of release, 4 relocations, top right), Vewe (10 years old at time of release, 93 relocations, bottom left) and Alex (12 years old at time of release, 100 relocations, bottom right). Red dots represent locations where orangutans have been released. Blue dots represent orangutan relocations. Green lines represent individual orangutan home range boundaries (MCP 100%). Blue lines represent orangutan population home range boundaries (Kernel 99%). Grey square with black dot represents location of the FZS station. (TIF) [file pone.0215284.s006.tif]

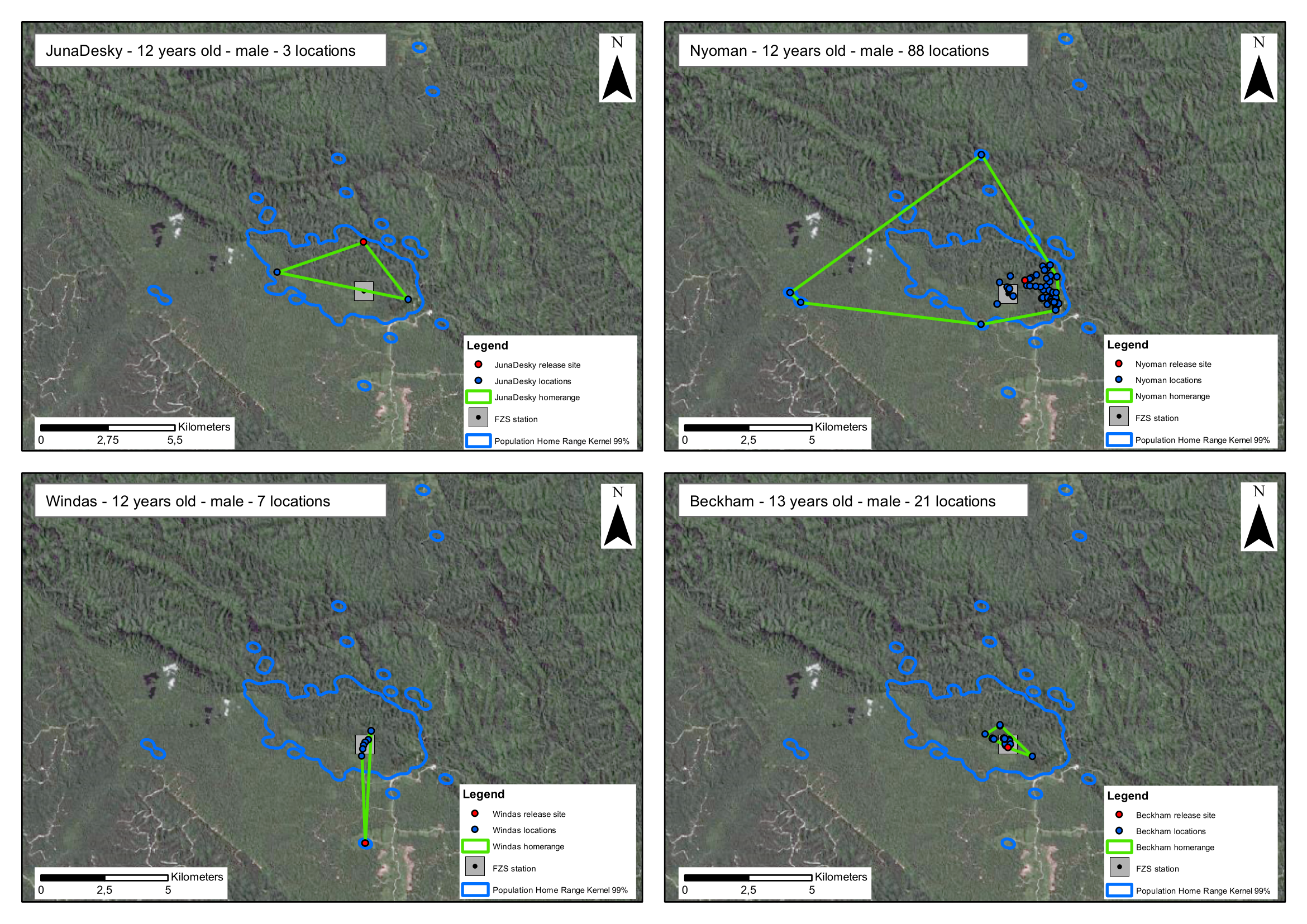

Supplement: S7 Fig — Individual maps for male orangutans JunaDesky (12 years old at time of release, 3 relocations, top left), Nyoman (12 years old at time of release, 88 relocations, top right), Windas (12 years old at time of release, 7 relocations, bottom left) and Beckham (13 years old at time of release, 21 relocations, bottom right). Red dots represent locations where orangutans have been released. Blue dots represent orangutan relocations. Green lines represent individual orangutan home range boundaries (MCP 100%). Blue lines represent orangutan population home range boundaries (Kernel 99%). Grey square with black dot represents location of the FZS station. (TIF) [file pone.0215284.s007.tif]

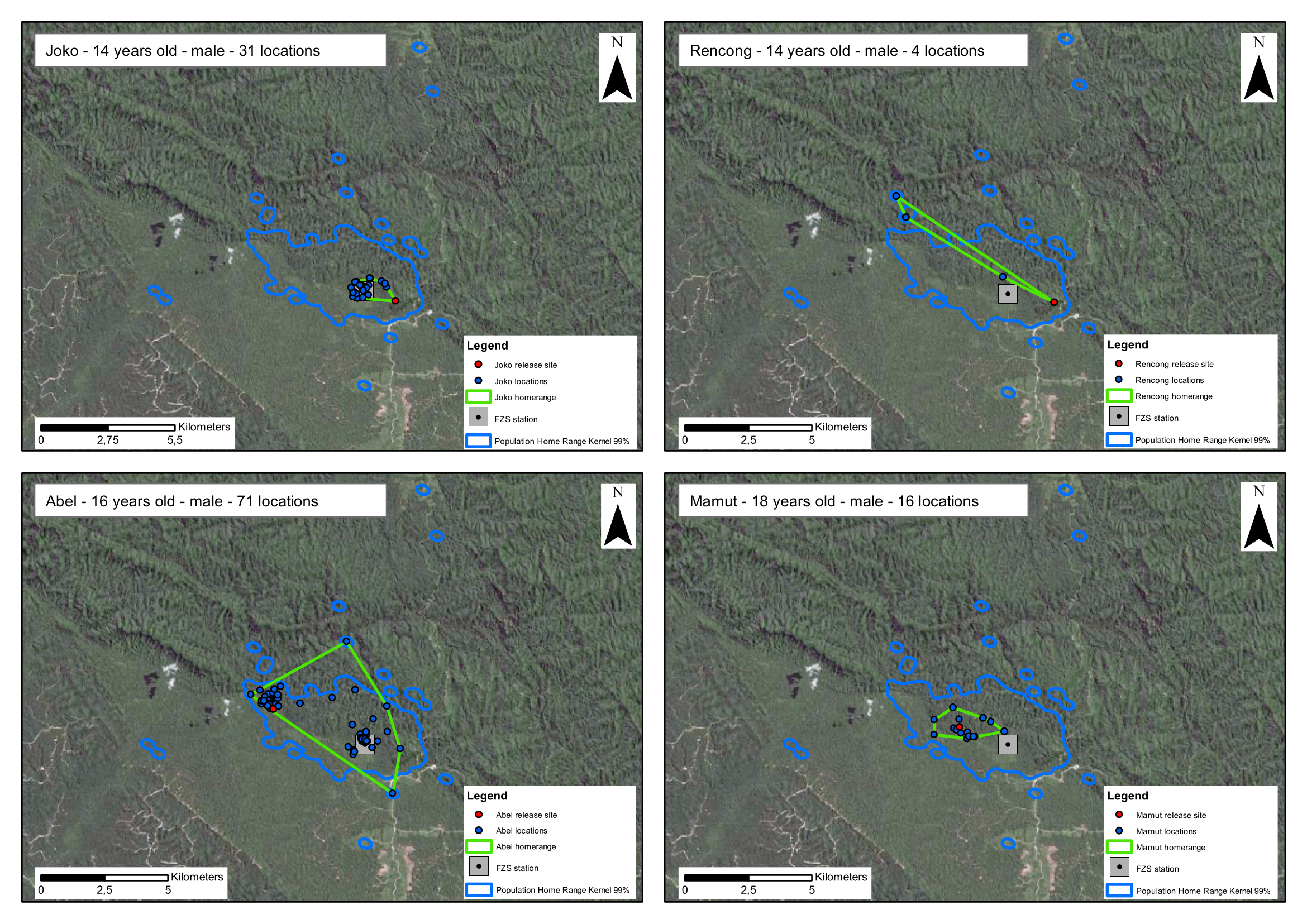

Supplement: S8 Fig — Individual maps for male orangutans Joko (14 years old at time of release, 31 relocations, top left), Rencong (14 years old at time of release, 4 relocations, top right), Abel (16 years old at time of release, 71 relocations, bottom left) and Mamut (18 years old at time of release, 16 relocations, bottom right). Red dots represent locations where orangutans have been released. Blue dots represent orangutan relocations. Green lines represent individual orangutan home range boundaries (MCP 100%). Blue lines represent orangutan population home range boundaries (Kernel 99%). Grey square with black dot represents location of the FZS station. (TIF) [file pone.0215284.s008.tif]

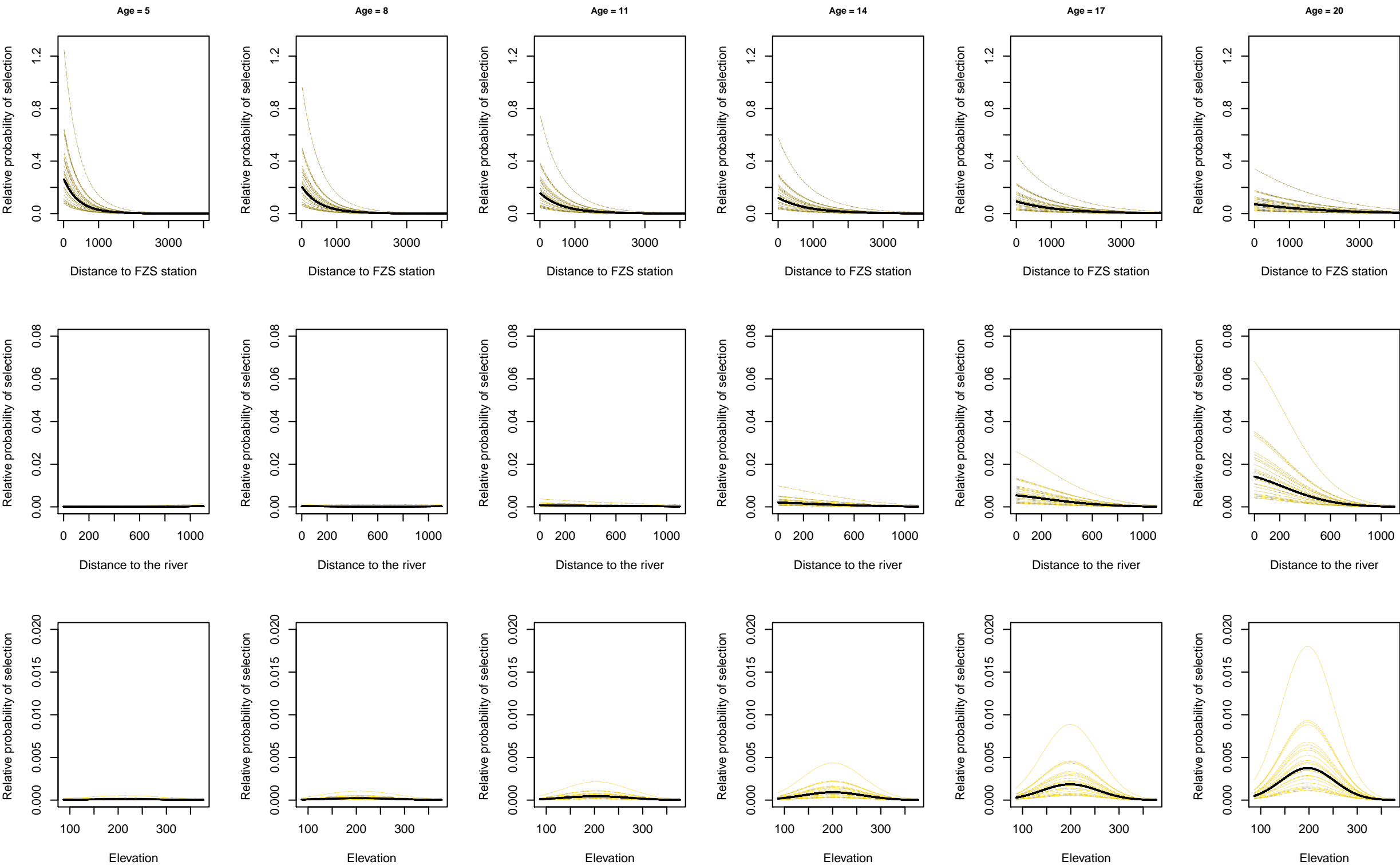

Supplement: S9 Fig — The black line represents the average value, other lines represent the parameter uncertainty related to inter-individual variability as predicted by the resource selection function. (PDF) [file pone.0215284.s009.pdf]

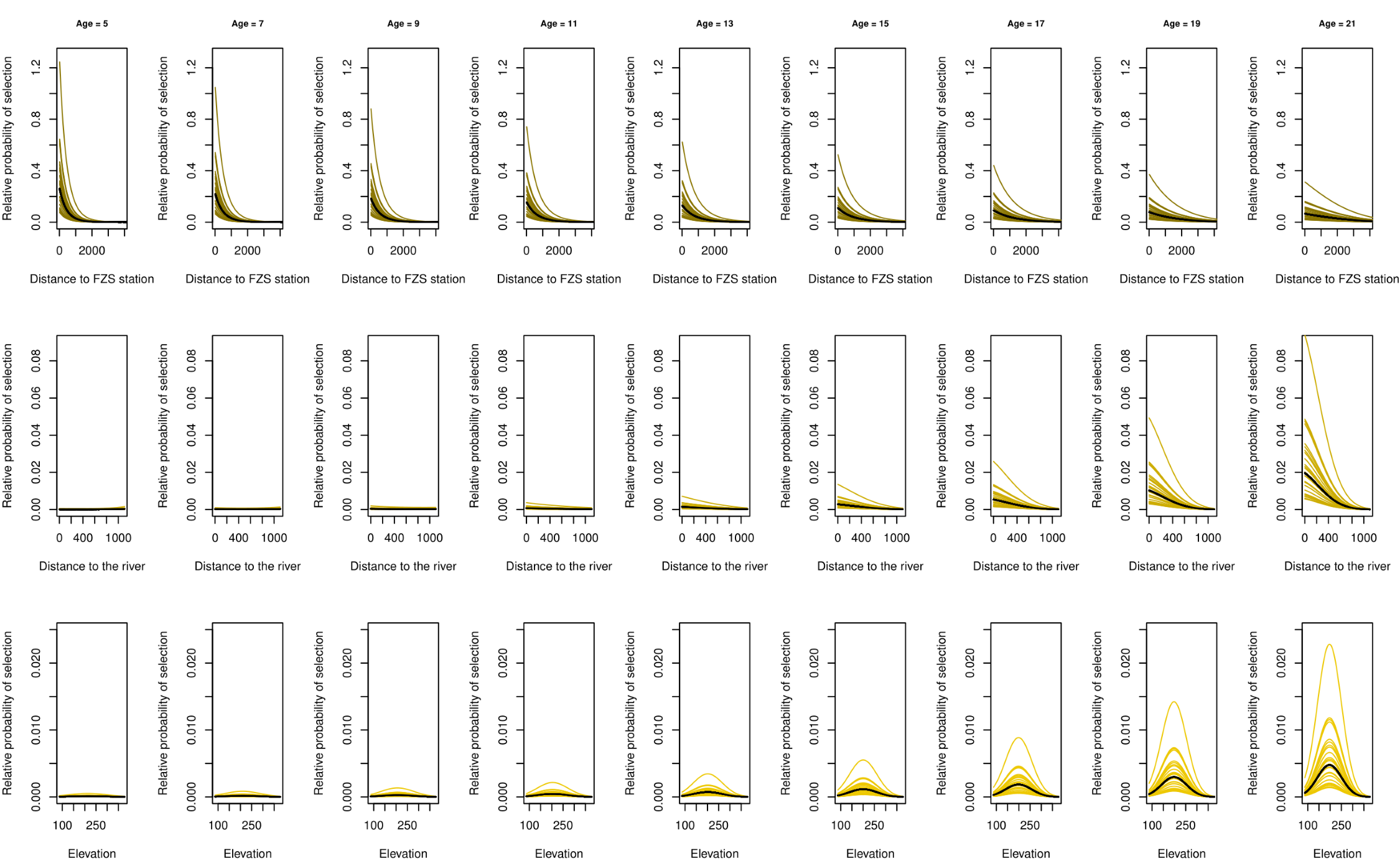

Supplement: S10 Fig — The black line represents the average value; other lines represent the parameter uncertainty related to inter-individual variability as predicted by the resource selection function. Age of monitored orangutans ranged from 5 to 21 years old, here depicted by nine age scenarios representing the evolution of resource selection patterns from younger to older individuals of the sample size. (TIFF) [file pone.0215284.s010.tiff]

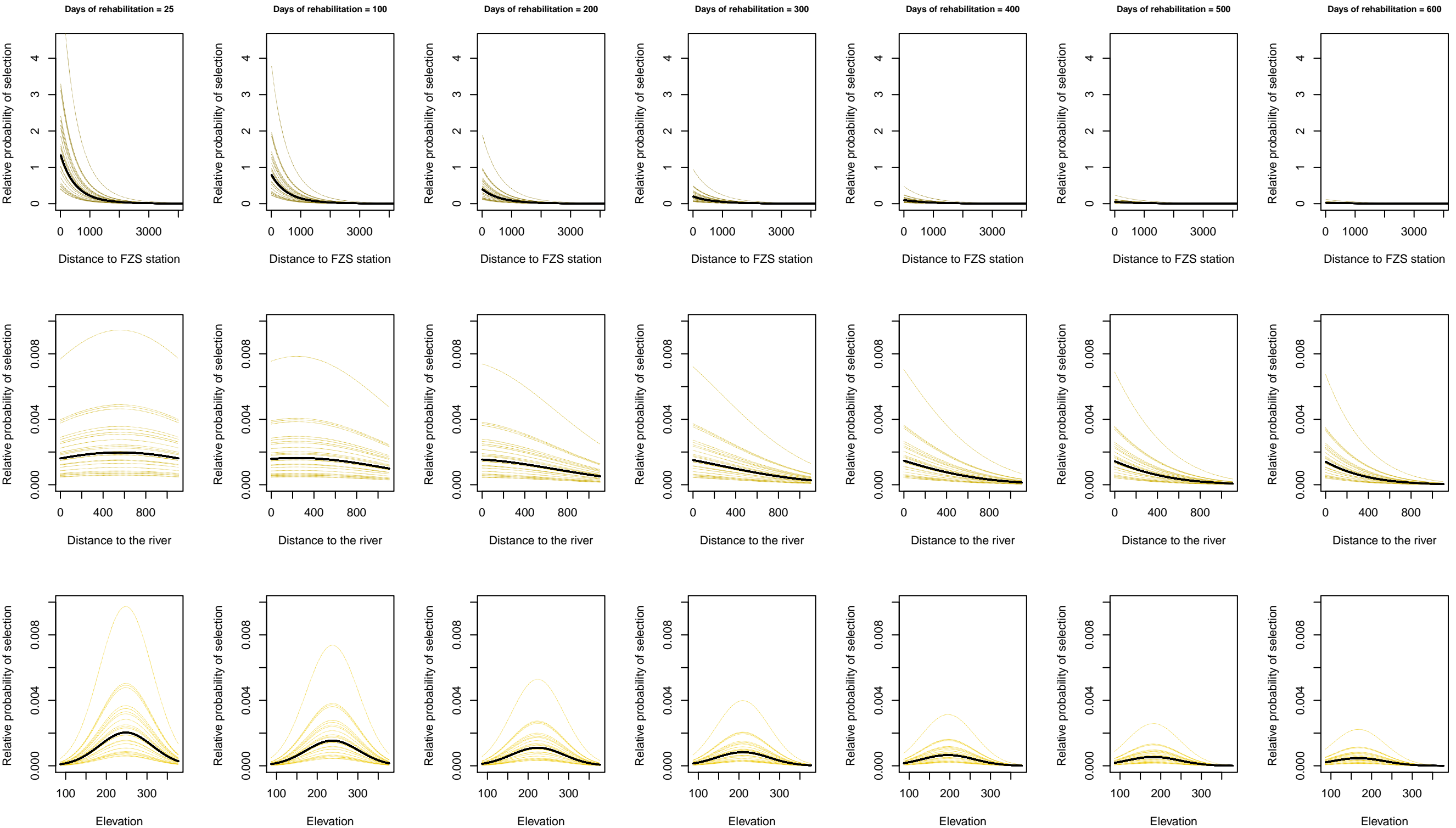

Supplement: S11 Fig — The black line represents the average value, other lines represent the parameter uncertainty related to inter-individual variability as predicted by the resource selection function. (PDF) [file pone.0215284.s011.pdf]

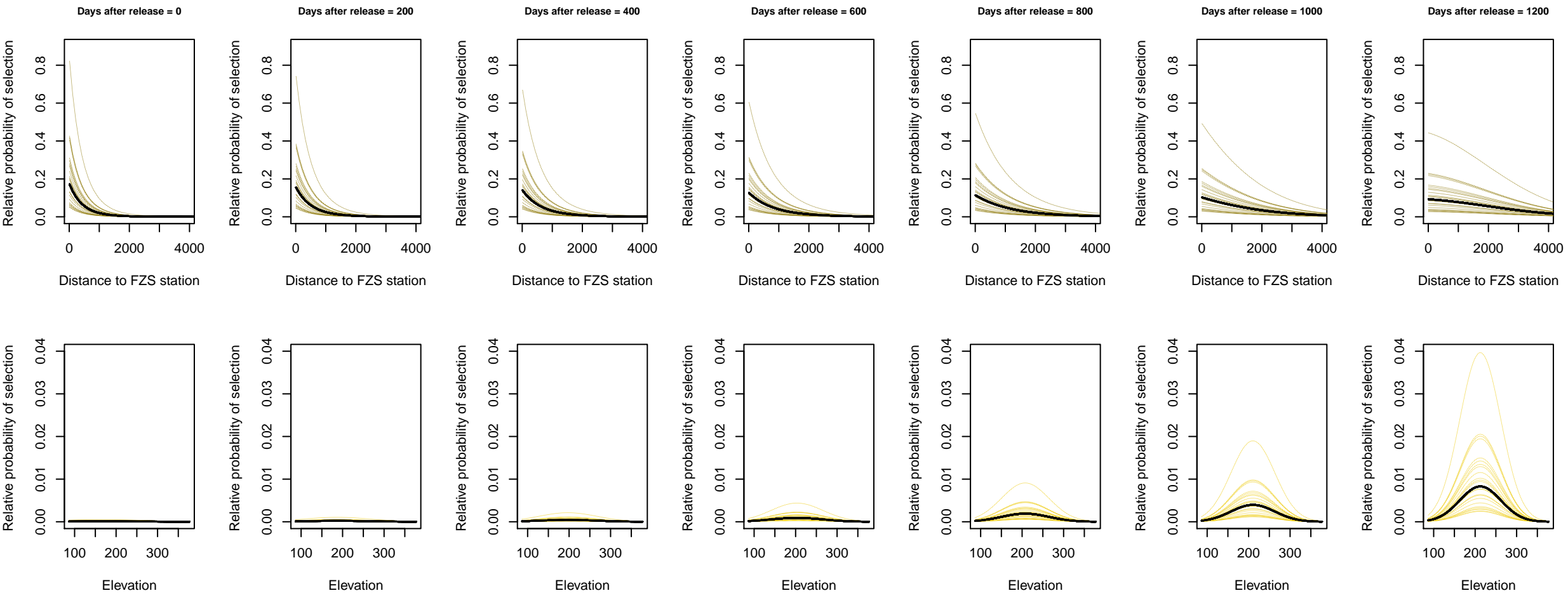

Supplement: S12 Fig — The black line represents the average value, other lines represent the parameter uncertainty related to inter-individual variability as predicted by the resource selection function. (PDF) [file pone.0215284.s012.pdf]

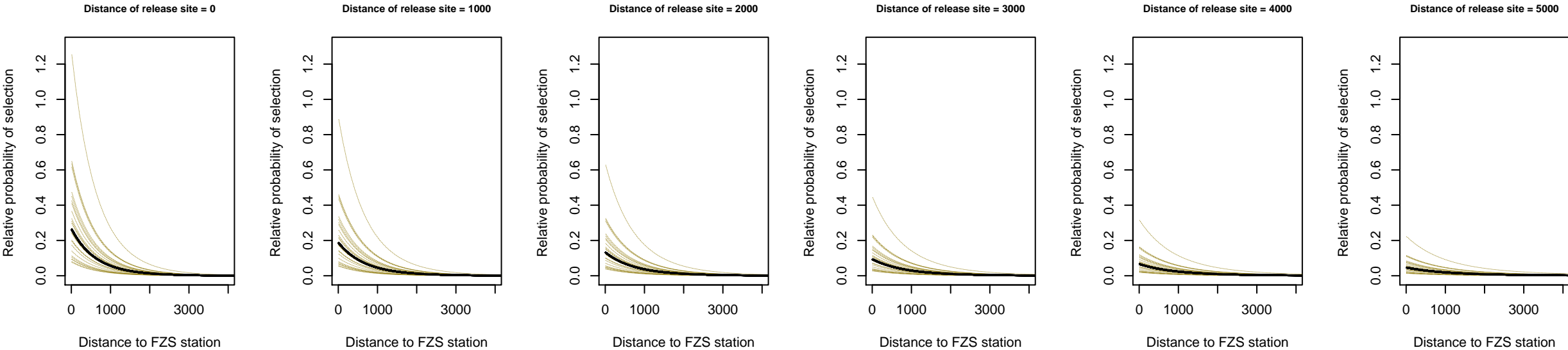

Supplement: S13 Fig — The black line represents the average value, other lines represent the parameter uncertainty related to inter-individual variability as predicted by the resource selection function. (PDF) [file pone.0215284.s013.pdf]
